# Tang Bi formula alleviates diabetic sciatic neuropathy via AMPK/PGC-1α/MFN2 pathway activation

**DOI:** 10.1038/s41598-025-10513-0

**Published:** 2025-07-11

**Authors:** Cunqing Yang, Honghai Yu, Linfeng Zhou, Hang Su, Xiangyan Li, Wenxiu Qi, Fengmei Lian

**Affiliations:** 1 Guang’anmen Hospital Affiliated to China Academy of Chinese Medical Sciences , Beijing, China; 2 Hunan University of Medicine General Hospital, Huaihua, Hunan China; 3 College of Traditional Chinese Medicine, Changchun University of Chinese Medicine, Changchun, Jilin China; 4 Northeast Asia Research Institute of Traditional Chinese Medicine, Key Laboratory of Active Substances and Biological Mechanisms of Ginseng Efficacy, Ministry of Education, Jilin Provincial Key Laboratory of Bio-Macromolecules of Chinese Medicine, Changchun University of Chinese Medicine, Changchun, 130117 Jilin China

**Keywords:** Diabetic peripheral neuropathy, UHPLC/QTOF, MS, AMPK, PGC, 1α, MFN2 pathway, Mitochondrial dynamics, Schwann cell, Diabetes, Neuroendocrine diseases

## Abstract

Diabetic peripheral neuropathy (DPN) is one of the most common chronic complications of diabetes mellitus, which affects various regions of the nervous system. Tang Bi formula (TBF) has been proven effective for DPN, while the underlying mechanism remains unclarified. This study aimed to clarifiy the neurprotective mechanism of TBF intervention in DPN through animal and cell models. UHPLC/QTOF-MS and network pharmacology analysis were utilized to identify the bioactive components and potential targets of TBF. DPN models were established in rats and Schwann cells to evaluate the therapeutic effects of TBF. In the DPN rats, body weight, fasting blood glucose, mechanical withdrawal threshold (MWT), paw withdrawal latency (PWL), sciatic motor nerve conduction velocity (MNCV), and sciatic nerve blood flow were measured. Pathological sections of the sciatic nerve (SN) were also examined. In vitro experiments, the Schwann cells (SCs) were cultured in a medium containing 30 mM glucose and treated with TBF for 48 h. Cell viability was assessed using the CCK-8 assay. The degree of apoptosis was evaluated by flow cytometry. The mitochondrial membrane potential was determined using JC—1 staining, and the generation of ROS was measured using DCFH—DA staining. Moreover, the expression levels of proteins related to the AMPK-PGC-1α-MFN2 pathway in the SN and SCs were detected. A total of 11 bioactive components of TBF were identified through UHPLC/QTOF-MS and network pharmacology analysis. In vivo experiments, MWT and PWL were decreased in DPN rats, which were restored after TBF administered daily for 12 weeks, TBF significantly attenuated thermal hyperalgesia and mechanical allodynia, and improved nerve conduction velocities. Further histopathological observations indicated that treatment with TBF promoted the regeneration of the myelin sheath of the SN, increased the density of intraepidermal nerve fibers, effectively improved distal microcirculation disorders, and alleviated demyelination and axonal degeneration. In vitro experiments were conducted to evaluate the protective effect of TBF on high-glucose-induced dysfunction of SCs. The data showed that treatment with TBF significantly inhibited the apoptosis of SCs. Meanwhile, TBF exhibited apparent antioxidant capacity, reducing the accumulation of intracellular ROS, and ameliorating mitochondrial dysfunction. Western blot analysis revealed that TBF activated the AMPK-PGC -1α-MFN2 pathway and upregulated the protein expressions of p-AMPK (Thr172), PGC-1α, and MFN2, suggesting that the neuroprotective effect of TBF was associated with the activation of this pathway. TBF ameliorated DPN by rectifying mitochondrial dynamic imbalance and modulating the activation of the AMPK-PGC-1α-MFN2 pathway. This, in turn, promoted neurogenesis and alleviated peripheral nerve lesions. Thus, this study demonstrated the therapeutic potential of TBF for DPN.

## Introduction

Approximately 50% of diabetic patients suffer from diabetic peripheral neuropathy (DPN)^1^. Sustained hyperglycemia induces progressive damage to sensory and motor neurons, manifesting as neuropathic pain, paresthesia, muscle weakness, and atrophy. In advanced stages, DPN can lead to foot ulcers and lower-limb amputations^2^. These complications not only severely impair quality of life but also impose substantial economic burdens on affected individuals^3^. Although glycemic control remains foundational for DPN management, longitudinal studies demonstrate that DPN prevalence persists at alarmingly high rates—even among patients with optimal glucose regulation. This suggests that hyperglycemic insult alone cannot fully explain DPN pathogenesis. However, all clinical trials attempting to alter the progression of DPN have so far ended in failure^4^. Therefore, there is an urgent need to identify effective therapeutic approaches to improve DPN.

A growing body of literature suggests that traditional Chinese medicine (TCM) may be a promising alternative treatment for polyneuropathy^5^. Herbs widely used in clinical practice exhibit significant neuroprotective, antioxidant, and anti-neuroinflammatory effects and ameliorate mitochondrial dysfunction, and they are effective at improving sensory–motor conduction velocities and neurologic function in DPN patients^5^. With multiple active ingredients and therapeutic targets, TCM, such as Astragali Radix (Name: Hedysarum Multijugum Maxim, Chinese name: Huangqi), Angelica sinensis (Name: Angelicae Sinensis Radix, Chinese name: Danggui), Panax notoginseng (Name: Panax Notoginseng, Chinese name: Sanqi), and *Ligusticum chuanxiong* (Name: Chuanxiong Rhizoma, Chinese name: Chuanxiong), exert significant curative effects toward DPN. Traditional medical studies have shown that wasting and thirsting disorders over time induce DPN, and it is believed that this disease is characterized by a concurrent deficiency in both qi and blood, along with inadequate blood circulation and venous stasis, leading to the impairment of venous structures and ligaments^6^. The neuroprotective and therapeutic effects of the traditional classic formula Huang qi Guizhi Wuwu Tang on DPN have been confirmed by multiple studies^7^. The Tang Bi formula (TBF) is derived from the classic traditional prescription Huangqi Guizhi Wuwu Tang with modifications and is believed to possess properties that nourish both qi and blood, enhance blood circulation while eliminating blood stasis, unblock the meridians, and provide pain relief. It has been shown to have therapeutic efficacy in improving the nerve conduction velocity (NCV) of DPN patients in the clinical setting. Nevertheless, the pharmacological mechanisms and active substances of TBF remain poorly understood and warrant further investigation.

Oxidative stress is considered as the core link in the occurrence and progression of DPN, which promotes disease progression by exacerbating mitochondrial dysfunction^8^. This study identified through network pharmacology analysis that the potential targets of TBF are associated with the Adenosine Monophosphate Activated Protein Kinase (AMPK) signaling pathway, which plays a crucial role in regulating mitochondrial function and reactive oxygen species (ROS) metabolism^8,9^. It is noteworthy that ROS, as an important regulator of mitochondrial dynamics^10^, its imbalance can disrupt the mitochondrial fission–fusion dynamic balance by regulating the phosphorylation of dynamin-related protein 1 (Drp1) and the expression of mitochondrial fusion proteins mitofusin 1 (MFN1), mitofusin 2(MFN2), and OpticAtrophyType1(OPA1). Abnormal mitochondrial dynamics have been confirmed to be involved in the pathological processes of neurodegenerative diseases such as diabetic peripheral neuropathy, Parkinson’s disease, and Alzheimer’s disease^11–13^, but the intervention mechanism of TBF in DPN remains unclear. This study first revealed that TBF can improve mitochondrial function by activating the AMPK/ peroxisome proliferator-activated receptor-γ coactivator 1α(PGC-1α) signaling axis, upregulating mitochondrial fusion-related proteins MFN1, MFN2, OPA1, and inhibiting Drp1 phosphorylation and mitochondrial translocation; meanwhile, TBF enhances AMPK pathway activity, inhibits Schwann cells (SCs) apoptosis and mitochondrial dysfunction, and alleviates nerve demyelination injury. This study reveals a novel mechanism by which TBF improves DPN through AMPK-mediated regulation of mitochondrial dynamics, providing a theoretical basis for traditional Chinese medicine compound intervention in neurodegenerative diseases.

## Materials and methods

### Preparation of TBF

Nine crude TBF drugs were provided by Guang’anmen Hospital of the Chinese Academy of Traditional Chinese Medicine (Beijing, China) at a fixed concentration of 9:15:3:9:9:15:9:3:15 (Supplementary Table 1). According to standard procedures, these nine medicines were decocted in 1000 mL of distilled water at 100 °C twice to obtain aqueous extracts. The extract was cooled and centrifuged (8,000 rpm, 10 m), after which the supernatant was collected. Finally, the powdered extract of TBF was obtained via freeze-drying. The final concentration was 0.1 g/ml, and the solution was dissolved in sterile PBS. TBF extract was diluted with culture media containing 10% Foetal Bovine Serum to the indicated concentrations.

### Analysis of the TBF formula and its medicated serum using UHPLC/QTOF-MS

A total of 45 standards were used in this experiment (Supplementary Table 2). UHPLC/QTOF-MS analysis was conducted using an Agilent 6550 mass spectrometer coupled with a UPLC system equipped with an ESI source (AGILENT, USA). We injected a total of 3 µL of the sample solution into an ACQUITY UPLC® BEH C18 column (2.1 × 100 mm, 1.7 µm Agilent Technologies, USA) for UPLC separation. The column temperature was maintained at 30 °C, and we performed gradient elution using a mobile phase consisting of 0.1% formic acid aqueous solution. (A) and 0.1% formic acid acetonitrile (B). The flow rate was set to 0.300 mL/min, and the maximum endurance was set to 800.0 bar.

For mass spectrometry (MS), the instrument was operated at drying gas and sheath gas temperatures of 200 ℃ and 350 ℃, respectively. The flow rates and settings during the analysis were as follows: the dry gas flow rate was set to 12 L/min, and the sheath gas pressure was maintained at 40 psi. The voltages applied were as follows: 390 V for the main voltage, 1,000 V for the nozzle voltage, and 3,500 V for the capillary voltage. The MS scan range was 100–1,500 m/z. The MS acquisition rates were 3 spectra/s with a dwell time of 333.3 MS/spectrum. MS/MS data analysis was performed using Mass Hunter Workstation Qualitative Analysis version 10.0 (Agilent).

The specific composition and chemical fingerprint of TBF were analyzed by an HPLC system with a 1260 DAD detector and a Chem Station chromatography workstation from Agilent Technologies, USA. The original fingerprint chromatograms from 10 batches of TBF complex samples were imported into fingerprint software for analysis.

### Network pharmacology analysis

Based on the Latin names of the targets of the blood-borne components of TBF obtained using UHPLC/QTOF-MS, the active targets of TBF were searched via the Swiss Target Prediction (http://www.swisstargetprediction.ch/). DPN-associated targets were identified via the Gene Cards (https://www.genecards.org/), OMIM (https://www.omim.org/), and Dis GENET (https://www.disgenet.org/) databases. The drug component targets, and disease targets were mapped to each other to produce Venn diagrams, resulting in the identification of intersecting genes. The resulting drug–target network was constructed using Cytoscape 3.7.2 software. The drug–disease intersection genes were uploaded to the String database (https://string-db.org/) for protein‒protein interaction network (PPI) analysis via Cytoscape 3.7.2. The DAVID database (https://david.ncifcrf.gov/summary.jsp) was used to annotate the role of target proteins in gene function for TBF treatment of DPN in terms of biological process (BP), cellular component (CC), molecular function (MF), and Kyoto Encyclopedia of Genes and Genomes (KEGG) pathway enrichment^14^. The top 10 BP, CC, and MF terms in the GO functional category and the 20 KEGG pathway entries related to DPN (*p* < 0.01) were selected as the main gene function enrichment process and signaling pathways for TBF treatment of DPN, and the mechanism of action of TBF treatment of DPN was predicted.

### Animal experiment

Adult male Sprague–Dawley (SD) rats, with an initial weight of 200–220 g, were purchased from Changchun Yi Si Experimental Animals Technology Limited [SCXK (Ji) 2020–0002, Changchun, Jilin, China]. This research was approved by the Biomedical Ethics Committee of Changchun University of Chinese Medicine (approval number no. 2023011). All experiments were performed in accordance with relevant guidelines and regulations. The rats were housed in the Animal Experimental Center at Changchun University of Traditional Chinese Medicine (Changchun, China) at 18–22 °C under a 12 h light–dark cycle. The rats underwent adaptive feeding for one month prior to the experiment. Then, they were randomly assigned to two groups: one control (CTRL) group (n = 10) and one experimental group (n = 50). After 12 h without food, the control group received an intraperitoneal injection of sodium citrate buffer solution. Moreover, the experimental group was administered an intraperitoneal injection of streptozocin (STZ) (65 mg/kg) dissolved in 0.1 M sodium citrate buffer (pH of 4.5). Three days later, we collected blood samples from the tail vein of the rats and measured their fasting plasma glucose (FBS) levels using an Accu-Chek Guide blood glucose meter (Roche, China). Rats with FBS levels less than 16.7 mM were excluded from this experiment. To determine the diabetic status of the rats with STZ, we measured their body weight weekly and FBS levels every two weeks.

Based on the FBS results, ten rats in the experimental group were excluded, and the remaining rats (n = 40) were allocated randomly into five groups (n = 8 rats/group) as follows: STZ group: pre-established DPN rats treated with 1.5 ml 0.9% saline by gavage; TBF groups: pre-established DPN rats treated with TBF (0.28 g/kg/d, 0.56 g/kg/d, 1.12 g/kg/d); and α-lipoic acid (ALA) group as a positive control: pre-established DPN rats treated with ALA (0.054 g/kg/d). The middle dosage of TBF used was based on the clinical dosage. The daily dosage for the rats was determined based on the pharmacological experimental methodology utilizing a conversion factor of 6.3 times the equivalent dose extrapolated from human-to-animal scaling.

Three normal SD rats were randomly selected and anesthetized by sodium pentobarbital intraperitoneal injection at 2, 4, and 8 h after drug administration, and blood samples were obtained from the abdominal aorta. After the serum was collected, a 50 µL sample was taken and mixed with 200 µL of methanol. The mixture was vortexed and then centrifuged at 12,000 rpm for 10 min to obtain the supernatant. Subsequently, the incoming blood components were analyzed by UHPLC/QTOF-MS.

### Paw withdrawal threshold (PWT) and mechanical hyperalgesia assessment (MWT)

The PWT and MWT were measured using an XR1700-Hot & Cold Plate Analgesia device and an XR-XZD-Rat and mouse analgesia meter. Briefly, rats were allowed approximately 15 min to adapt to the assessment chamber before testing, after which they were positioned on a hot plate at 55 °C or separately placed in three cages to stimulate the left hind paws with a mechanical needle. We defined a positive response as the licking of paws and skipping and stamping (cutoff period set to 10 s) or pressure exerted using the metal probe. The average value was recorded as the latency period^15^.

### Measurement of the sensory nerve conduction velocity (SNCV) and motor nerve conduction velocity (MNCV)

According to a previously described method, we determined the SNCV and MNCV of the sciatic nerve (SN)^16^. Briefly, rats were placed in the prone position, and a stimulating electrode was placed on the proximal end of the SN (recorded as the S channel), while a recording electrode was positioned on the muscular abdomen of the gastrocnemius along the path of the SN (recorded as the R channel). A reference electrode needle was located between the two recording electrode needles close to the recording point. A single-pulse square wave stimulus with a pulse width of 0.206 ms, a maximum repetition rate of 2 Hz, a scanning speed of 50 ms/D, and a stimulus intensity of 2 mA was used. The NCV was measured using a PL-3508 Data Acquisition and Analysis System (AD Instruments, New Zealand).

### Regional blood flow perfusion measurement

After 12 weeks of treatment, regional blood flow perfusion was assessed using a previously described method ^17^. The blood flow of the SN and plantar skin was measured using laser speckle contrast imaging (LSCI).

### Morphological observation of the SN

The SN specimens were fixed with 4% formaldehyde solution. After the SN samples were sectioned to a thickness of 5 μm, hematoxylin and eosin (H&E) staining, glycine silver staining, Luxol fast blue (LFB) staining, and Nissl staining were used to assess the myelination status and neuronal damage. Furthermore, SNs were fixed overnight in a 2.5% glutaraldehyde solution in 0.1 M phosphate buffer (pH of 7.4) at 4 °C for transmission electron microscopy (TEM). Subsequently, they were subjected to postfixation for 2 h in a 0.1 M osmium tetroxide solution. Next, the specimens underwent dehydration and were embedded in epoxy resin. Ultrathin slices were then cut, stained with uranyl acetate and lead citrate, and photographed and analyzed using a HITACHI TEM-HT7700 TEM from Japan.

### Western blots analysis

Proteins were extracted from the SN tissues of rats and Schwann cells, and the protein concentration was subsequently measured with a BCA protein assay kit (Beyotime, China). Subsequently, SDS‒polyacrylamide gel electrophoresis was performed, and the membranes were then exposed to primary antibodies, including anti-phosphorylation-DRP1 (Ser616, 1:1000, Cell Signaling Technology), anti-DRP1 (1:1000, Proteintech), anti-mitochondrial fission protein 1 (FIS1) (1:2000, Proteintech), anti-MFN1 (1:500, Proteintech), anti-OPA1 (1:1000, Proteintech), anti- MFN2 (1:1000, Proteintech), anti-phosphorylated AMPK (Thr172) (1:5000, Abcam), anti-AMPK (1:1000, Abcam), and anti-PGC-1α (1:1000, Proteintech). The membranes were subsequently incubated with appropriate secondary antibodies. Notably, anti-β-tubulin (1:5000, Proteintech) served as a normalization control.

### Cell culture and treatment

Schwann cells (SCs) were purchased from the National Infrastructure of Cell Line Resource (Beijing, China). The cells were cultured in Dulbecco’s modified Eagle’s medium with low glucose (DMEM, Sigma, USA) supplemented with 5.6 mM glucose, 1% penicillin and streptomycin, and 10% fetal bovine serum (Clark Bioscience, USA). The cells were cultured in a thermostatic chamber (5% CO_2_, 37 °C). After the cells reached 60–70% confluence, the SCs were treated with 30 mM glucose to construct an HG-induced cell model. Then, the SCs were separated into six groups, including the following: the normal glucose (NG) group in which SCs were grown in normal glucose (5.6 mM glucose) medium; the high glucose (HG) group in which SCs were grown in high glucose (30 mM glucose) medium; the TBF group in which SCs were grown in TBF-treated HG medium (HG + 50, 100, 200 μg/mL TBF); and the AICAR (AMPK activator) group in which SCs were grown in AICAR-treated HG medium (HG + 0.5 mM AICAR).

### Cell viability assay

Cell proliferation was evaluated by the Cell Counting Kit-8 (CCK-8) assay (Beyotime Biotechnology, China). SCs were plated in 96-well plates at 3 × 10^3^ cells per well. To investigate the impact of HG on cell viability, the cells were cultured in HG medium for 24, 48, and 72 h. Subsequently, the cells were divided into the NG group, HG group, and different concentrations of TBF-treated HG medium (50, 100, 200, 400, 800, and 1000 μg/mL) for 24, 48, and 72 h to examine the effects of TBF on the viability of HG-treated cells. Cell viability was expressed as (A_experimental_–A_blank_)/(A_control_–A_blank_) × 100%. The cell viability of the NG group was set as 1, representing the baseline.

### ROS measurement

ROS were quantified with an ROS assay kit (Beyotime Biotechnology, Shanghai, China). Briefly, the cells were exposed to 10 μM 2’,7’-dichlorofluorescin diacetate (DCFH-DA) at 37 °C for 30 min, and flow cytometry (Beckman Coulter, Inc., USA) was used to quantify the ROS levels.

### Mitochondrial membrane potential (MMP) determination

To assess the MMP, we used a JC-1 assay kit (Beyotime Biotechnology, China). First SCs were incubated with JC-1 for 20 min at 37 °C in the dark. After two washes, the ratio of red (aggregation) to green (monomer) fluorescence intensity was assessed by examining the cells under a fluorescence microscope. A decrease in the MMP was indicated by a decrease in the ratio of red (aggregation) to green (monomer) fluorescence intensity.

### Cell apoptosis assay

SCs were harvested and labeled with Annexin V and propidium iodide (PI) using an apoptosis kit (Key Gen Biotech, China) and analyzed using a flow cytometer.

### Statistical analysis

All the statistical analyses were performed using GraphPad Prism 9.4.1 and SPSS 26.0. We performed the Shapiro Wilk test and Q-Q plots for assessing normality. All the data were expressed as mean ± SEM. For the comparison between two groups, Student’s t-tests were used considering the normally distributed data. Among multiple groups, comparisons were performed using one-way analysis of variance (ANOVA) on the normally distributed data, the least significant difference (LSD) tests were used for post hoc analysis. The Kruskal–Wallis H-test was used when the data had a nonnormal distribution, then the Dunn’s pairwise comparison test was carried out as a post hoc test. Values of *P* < 0.05 were considered statistically significant.

## Results

### Chemical profiling of TBF

**Fig. 1:**
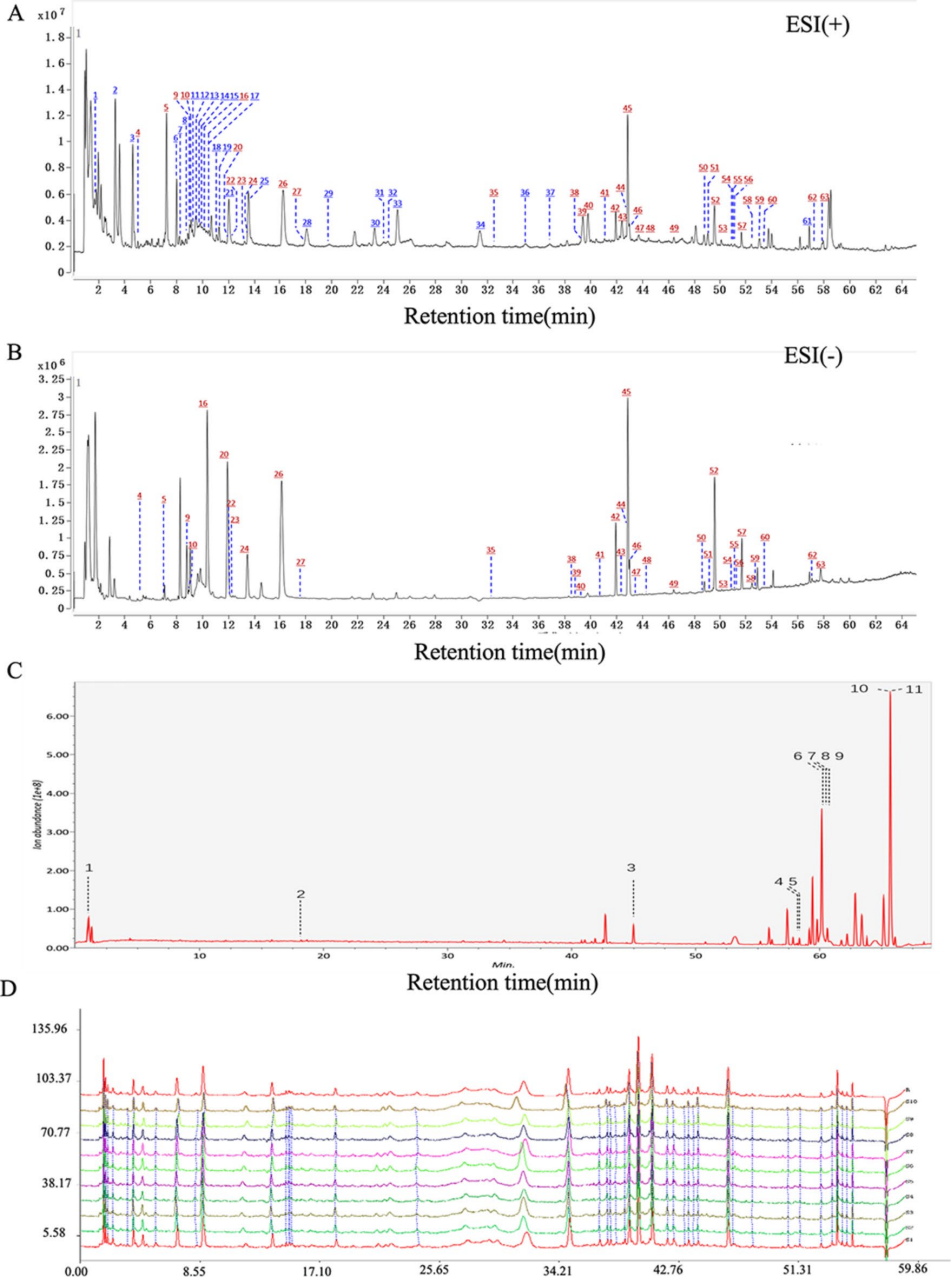
The UHPLC/QTOF-MS of TBF solution and serum sample. (**A**) The HPLC chromatogram of TBF solution in positive ion mode. (**B**) The HPLC chromatogram of TBF solution in negative ion mode. (**C**) The HPLC of serum sample collected from rats by gavage administration of TBF solution. (**D**) The HPLC fingerprints of 10 batches of TBF solution.

TBF solution and serum sample solution were identified via UHPLC/QTOF-MS analysis. After optimizing the UHPLC and MS conditions, base peak ion chromatograms were obtained in positive-ion and negative-ion modes, demonstrating excellent separation. A total of 63 compounds were identified in the TBF solution (Supplementary Table 3). Among these, 11 were absorbed into the blood (Fig. 1A-C), and their chemical identification data (retention time, m/z, molecular formula, and MS/MS spectra) are provided in Supplementary Table 4. These compounds included nicotinamide, 2,2’,5’-trihydroxy-4-methoxy chalcone, albiflorin, 4-hydroxy-3-butylphthalide, 3-(4-ethylbenzoyl) propionic acid, hesperidin, sappanone B, protosappanin C, brazilein, senkyunolide, and senkyunolide G (Fig. 1C). All 63 identified compounds can be found in single herbal medicines. Furthermore, detailed identification information for these compounds is provided in Supplement 3. The fingerprint spectra of 10 batches of samples were compared with the generated reference spectrum. The similarity of each sample ranged from 0.858 to 0.950 (Fig. 1D). The results indicate that the first, fifth, sixth, and tenth batches of samples showed lower similarity compared to the reference fingerprint spectrum. This could be due to differences in the geographical conditions, harvesting time, and processing methods of individual herbal ingredients in different batches of the formula, leading to variations in the similarity of their fingerprint spectra.

### Network pharmacology study of the components of TBF in rat serum

**Fig. 2:**
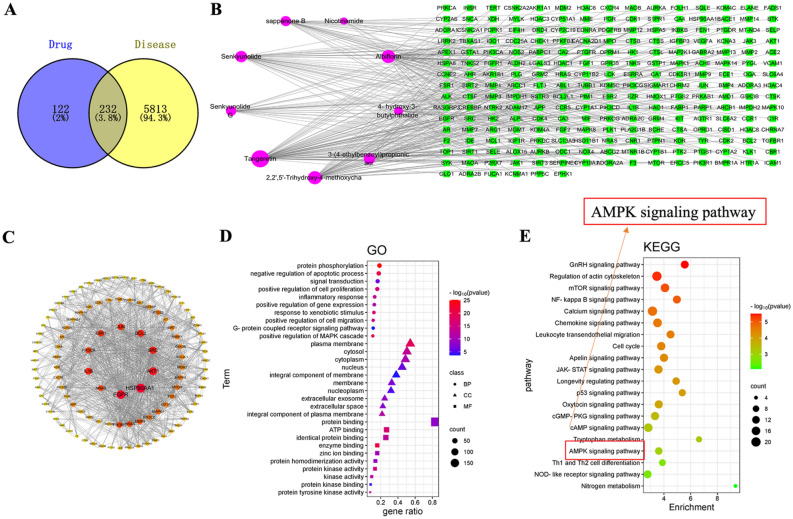
Network analysis. (**A**) Venn diagram of intersecting targets of TBF against DPN. (**B**) The network diagram of “Drug-disease-component-targets”. (**C**) PPI network. (**D**) GO enrichment analysis. (**E**) KEGG pathways analysis. The figure is based on the KEGG pathway map (Kanehisa et al., 2019; available at www.kegg.jp/kegg/kegg1.html).

The 11 compounds absorbed from rat blood were identified as potential bioactive components of TBF, and their potential targets were predicted using a network pharmacology approach. We identified 6,045 target genes associated with DPN and 354 TBF targets. By intersecting the target genes of the drug with those of DPN, we identified 232 intersecting target genes that represent the interactive target genes for DPN drug therapy (Fig. 2A). Using these targets, we generated a network diagram illustrating the anti-DPN effects of TBF (Fig. 2B). The 232 obtained intersecting target genes were imported into Cytoscape 3.7.2 software to visualize the protein‒protein interaction network (Fig. 2C). The key targets included HSP90AA1, EGFR, AKT1, SRC, IL1B, BCL2, JUN, ESR1, PIK3CA, and NRAS (Fig. 2C). Apoptosis is related to the key targets HSP90AA1 and BCL2 and to the mitochondria-dependent pathway. Notably, 232 targets were subjected to GO and KEGG enrichment analysis via the DAVID database. As shown in Fig. 2D, GO enrichment analysis of key targets revealed that the active ingredients may primarily regulate protein phosphorylation, cellular apoptosis, signal transduction, and inflammation by affecting mitochondrial ATP synthesis, exerting their therapeutic effects on DPN. These findings underscore the importance of mitochondria in DPN and provide crucial clues for further elucidating the therapeutic mechanism of TBF.

Through KEGG enrichment analysis and literature screening, pathways related to DPN were identified (Fig. 2E). The results showed that key target genes may be significantly associated with biological pathways and signaling pathways such as the mTOR signaling pathway, NF-κB signaling pathway, calcium signaling pathway, chemokine signaling pathway, JAK-STAT signaling pathway, p53 signaling pathway, cAMP signaling pathway, and AMPK signaling pathway. These results suggest that the main components of TBF may exert therapeutic effects on DPN by regulating key pathways. Notably, the AMPK signaling pathway was predicted to be one of the most important signaling pathways.

### TBF ameliorates metabolic derangements and neurological deficits in DPN rats

**Fig. 3:**
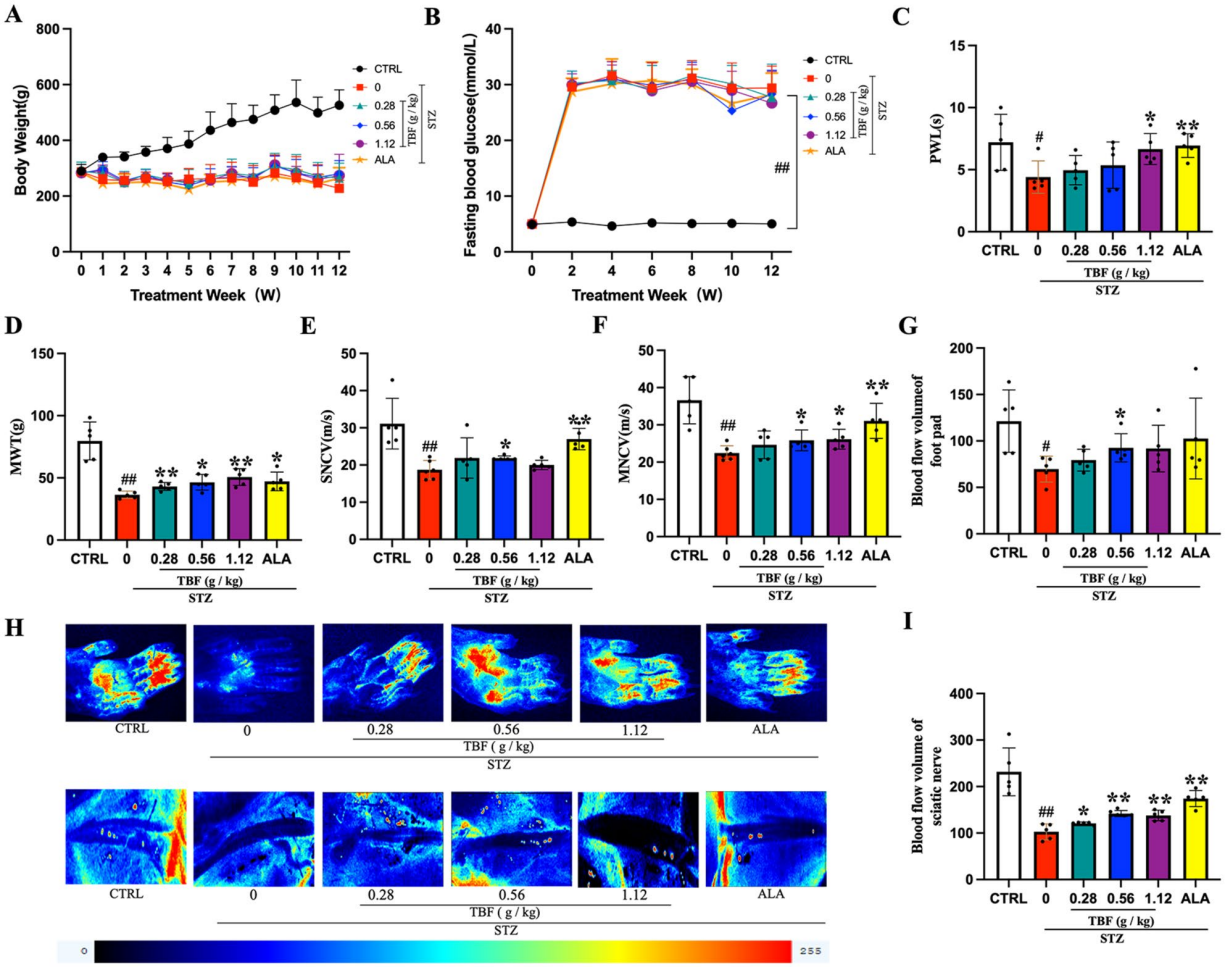
TBF improved DPN-related phenotypic characteristics and neurological functions in rats. (**A**) Body weight. (**B**) FBS. (**C**) PWL in thermal stimulation. (**D**) Plantar recession thresholds under MWT. (**E**) MNCV. (**F**) SNVC. (**H**) Representative images of regional perfusion volumes in the footpad tissue and SN of the rats. (**G, I**) Quantitative analysis of regional blood perfusion in footpad tissue and SN. Blue represents minimum blood flow, while red represents maximum blood flow, and green or yellow represents intermediate levels. N = 5 in each group, #*p* < 0.05; ##* p* < 0.01, versus. CTRL group. * *p* < 0.05; ** *p* < 0.01 versus. STZ group.

To assess the systemic and neurological impacts of TBF, we induced a diabetic rat model using STZ. DPN is frequently associated with metabolic dysregulation and progressive neurological decline. As illustrated in Fig. 3A, the body weights of CTRL—treated rats increased with age. In contrast, compared to the CTRL group, the body weights of rats in the STZ group declined one week post—injection. Hyperglycemia led to the consumption of fat and protein in rats, indicative of hyperglycemia—induced catabolism.

Prior to STZ injection, the FBS level in the CTRL group remained stable at 5.05 ± 0.55 mmol/L, while in the experimental group, it was 5.02 ± 0.41 mmol/L. After STZ injection, the STZ group exhibited a significant elevation in FBS levels compared to the CTRL group (Fig. 3B). Notably, during treatment, the FBS levels in the TBF and ALA groups did not differ significantly from those in the STZ group (Fig. 3B), suggesting that TBF’s neuroprotective effects are independent of glucose regulation.

The progression of DPN was quantified by measuring PWL and MWT. PWL and MWT were decreased in STZ-induced rats. The latency times in the TBF and ALA groups were longer than those in the STZ group, indicating an improvement in pain perception and hyperalgesia (Fig. 3C, D). NCV serves as a gold—standard parameter for diagnosing DPN and evaluating therapeutic efficacy. Thus, we measured NCV at the 12—week mark of treatment. As depicted in Fig. 3E, F, compared to the CTRL group, the SNCV of rats in the STZ group were significantly lower (31.12 ± 6.81 vs. 19.98 ± 2.89 m/s), as were then MNCV (36.61 ± 6.32 vs. 22.42 ± 1.98 m/s), signifying the onset of peripheral neuropathy. However, 12—week repeated administration of TBF and ALA led to substantial improvements in SNCV and MNCV compared to the STZ group (Fig. 3E, F).

LSCI revealed decreased blood flow in the plantar skin and sciatic nerve of STZ rats, reflecting DPN—associated microangiopathy. TBF treatment significantly improved this reduced blood flow (Fig. 3G, H, I). The positive—control drug ALA also enhanced blood flow in the sciatic nerve of STZ—induced rats (Fig. 3H, I). Interestingly, there was no significant difference in the improvement of plantar skin blood flow between the ALA- treated group and the STZ group (Fig. 3G, H). These findings suggest that TBF holds potential for enhancing neurological function in STZ—induced diabetic rats.

### Administration of TBF prevents STZ-induced SN injury in rats

**Fig. 4:**
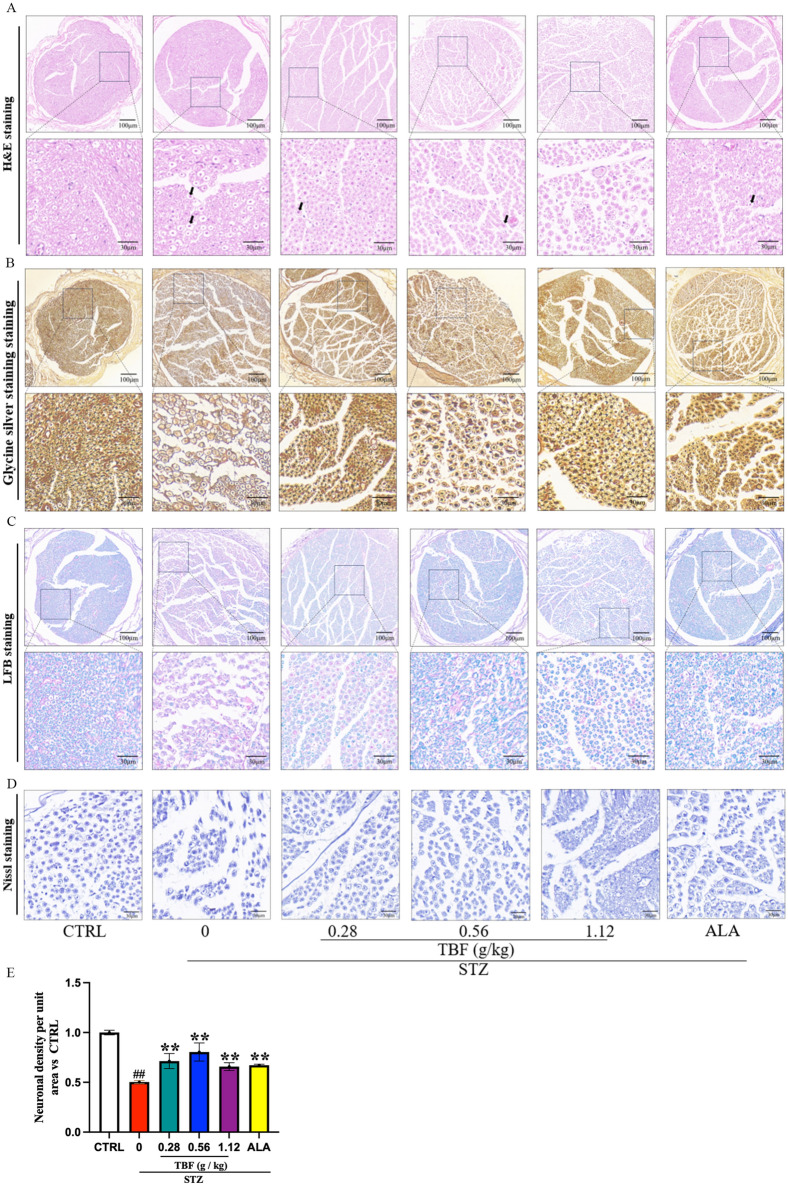
H&E, Glycine silver, LFB and Nissl staining for observing pathologic changes of SN tissues. (**A**) H&E staining. (**B**) Glycine silver staining. (**C**) LFB staining. (**D**) Nissl staining. (**E**) Quantification of neuronal density per unit area in Nissl staining, N = 3 per group. #*p* < 0.05; ##*p* < 0.01, versus. CTRL group. *p < 0.05; **p < 0.01, versus. STZ group.

DPN is associated with myelin sheath changes and axonal fiber degeneration^18^. To further determine the protective impact of TBF on STZ-induced rats, we performed H&E, glycine silver, LFB and Nissl staining (Fig. 4). In the CTRL rats, the morphology and structure of the SN fibers were normal, and the myelinated nerve fibers were arranged in an orderly manner. In the STZ group, more swollen and vacuolated nerve fibers were observed (Fig. 4A). Glycine silver staining was used to evaluate the degree of nerve damage in the rats (Fig. 4B), and the SN fibers exhibited axonal swelling, vacuolation, and degeneration in the STZ group. LFB staining revealed that the SN of the STZ group exhibited darker myelin sheath staining and changes such as swelling, tortuous and spherical structures, and fragmentation (Fig. 4C). Compared to those in the STZ group, the rats in the TBF and ALA groups exhibited milder pathological changes, as mentioned above. Nissl staining results demonstrated that the STZ rats exhibited fewer blue-stained Nissl bodies. The TBF and ALA groups of rats showed more blue-stained Nissl bodies than did the STZ group, with increased neuronal density within the unit area (Fig. 4D, E).

### TBF improved the ultrastructure of the SN in STZ-induced rats

**Fig. 5:**
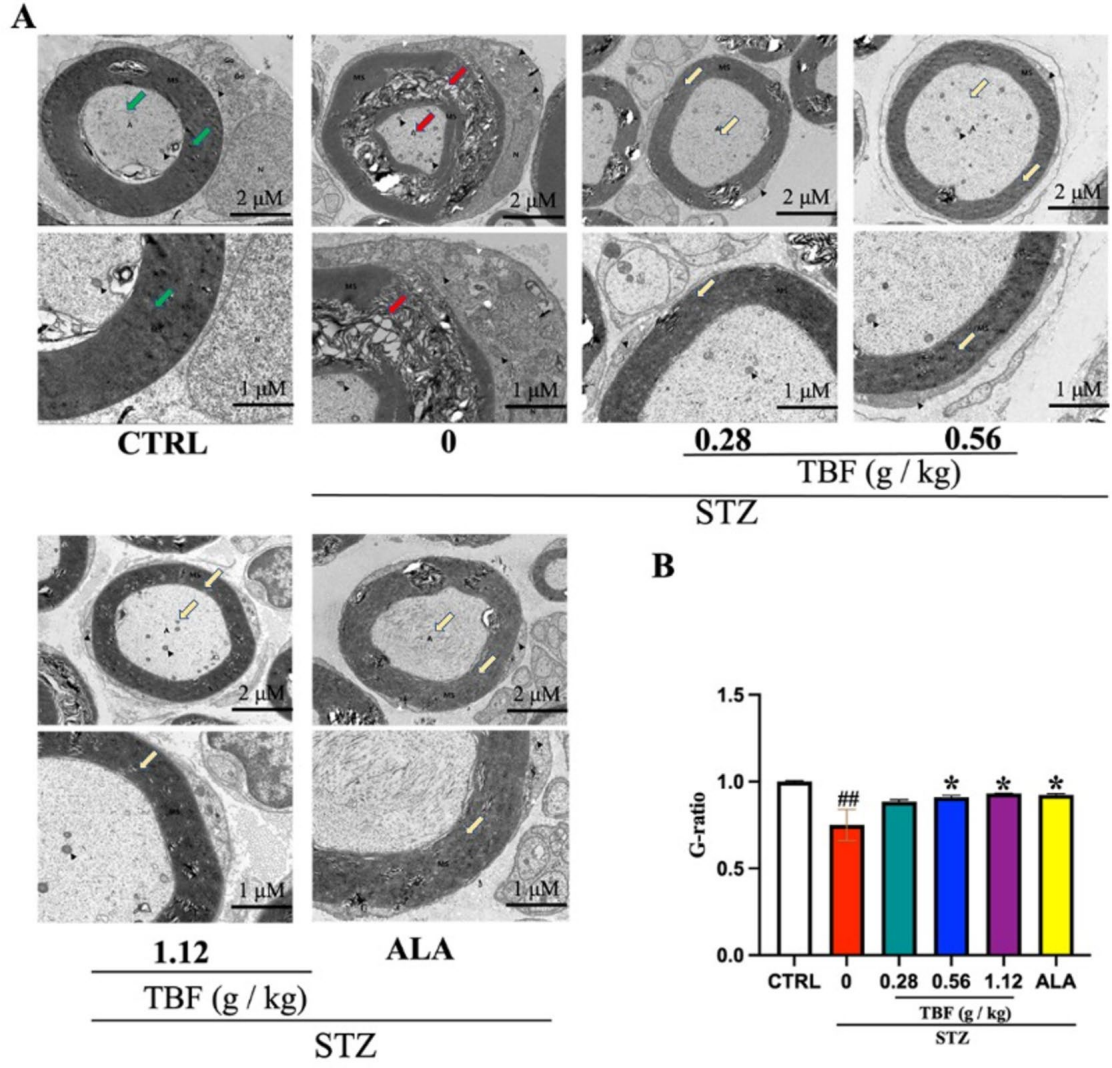
The ultrastructure of SN tissues was observed using TEM. (**A**) Representative images of the SN obtained by TEM. Panels show cross-sectional views at 4000x (2 μM) and 8000x (1 μM) magnification. (**B**) G-ratio measurement in the SN. Data was expressed as mean ± SEM, n = 3 for each group. #*p* < 0.05; ##*p* < 0.01, versus. CTRL group. *p < 0.05; ** *p* < 0.01 versus. STZ group. A: axon; MS: myelin sheath; N: nucleus; Black arrowhead: mitochondria; Nu: nucleolus. Red arrows indicate: myelin sheath damage (lamellar structure lysis, dispersion), axonal degeneration (reduced axonal diameter); green arrows mark normal structures; yellow arrows denote: myelin sheath and axonal repair.

TEM was employed to investigate the impact of TBF on the ultrastructural alterations of the SN in rats with diabetes mellitus induced by STZ. In the CTRL group, myelinated nerve fibers were enveloped by lamellar myelin sheaths. However, in the STZ group, the diameter of the axons in the SN decreased, and significant damage to the myelin sheath was observed. The laminar structure of the myelin sheath was dissolved, and there were signs of discrete and phasic destruction, along with locally visible demyelination. Additionally, the mitochondria were significantly swollen, with fewer cristae. Treatment with TBF or ALA improved myelin demyelination and effectively corrected axonal atrophy (Fig. 5A). Furthermore, the G-ratio (axon diameter/fiber diameter), a key indicator of myelin integrity, was markedly lower in the STZ group than in the CTRL group (Fig. 5B). The G-ratio was notably greater in the TBF and ALA groups than in the STZ group (Fig. 5B). These findings revealed that TBF significantly reversed STZ-induced neurotoxic defects in the SN, including axonal degeneration and myelin damage.

### Effects of TBF on the AMPK pathway and mitochondrial dynamics in the SN of STZ-induced rats

**Fig. 6:**
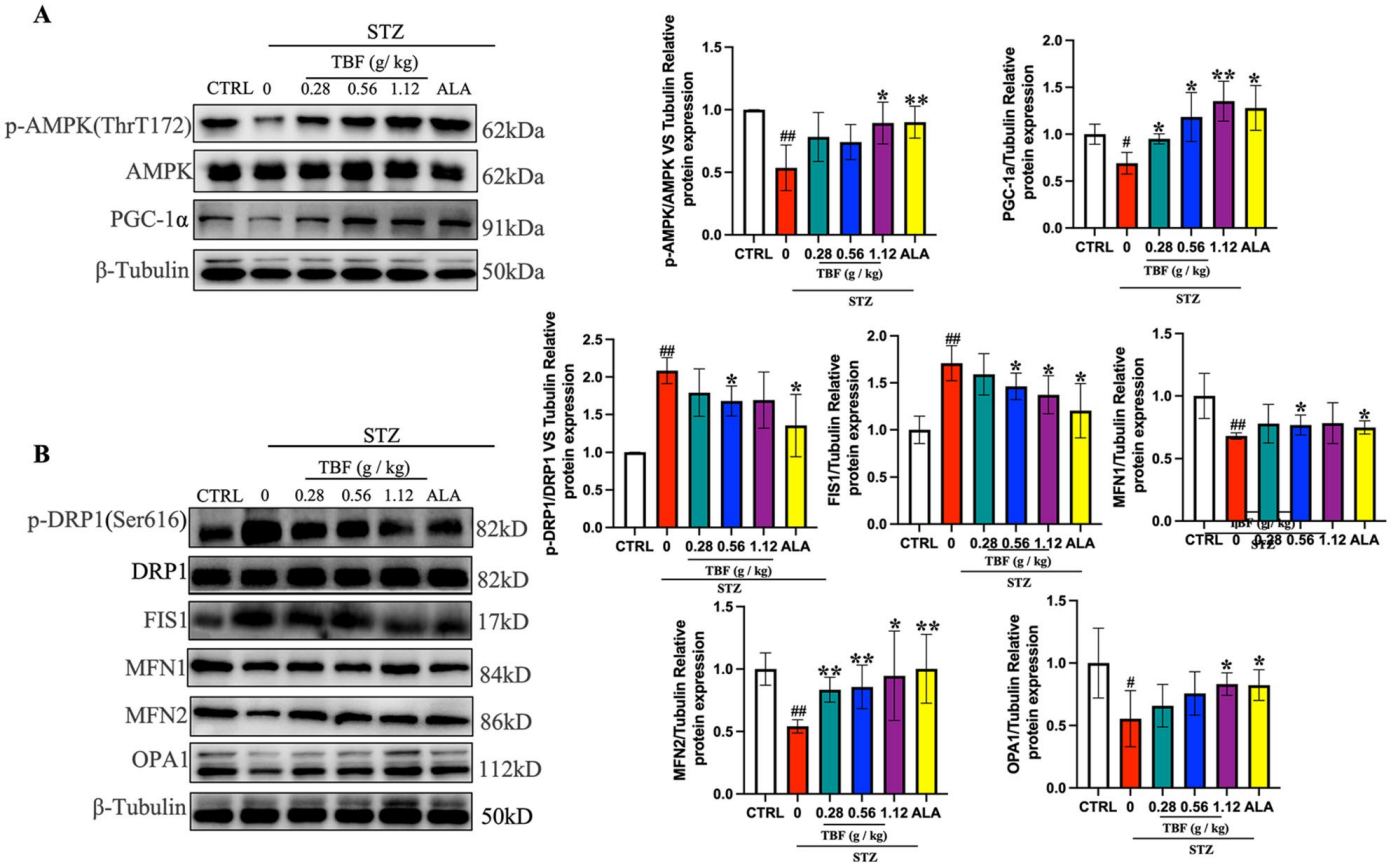
TBF regulated the AMPK pathway and the changes in its downstream mitochondrial dynamics of STZ-induced rats. (**A**) The expression levels of p-AMPK, AMPK, PGC-1α proteins in the SN were measured by western blotting. (**B**) The levels of mitochondria dynamics-relevant proteins in the SN were determined using western blotting analysis. The protein quantification results were reported as fold changes compared to the CTRL group. #* p* < 0.05; ##* p* < 0.01, versus. CTRL group. * *p* < 0.05; ** *p* < 0.01 versus. STZ group.

Based on the potential mechanisms predicted by previous network pharmacology studies and the current state of research, we examined the impact of TBF on the AMPK signaling pathway and changes in downstream mitochondrial dynamics. As shown in Fig. 6A, compared to those in the CTRL group, the STZ group exhibited decreased p-AMPK/AMPK and PGC-1α protein levels, indicating that the AMPK pathway was inhibited. However, after treatment with TBF and ALA, this inhibition was reversed. Notably, TBF decreased the levels of mitochondrial fission-related proteins in the SN of STZ-induced diabetes mellitus rats. The levels of p-DRP1 (Ser616)/DRP1 and FIS1 greatly decreased in the TBF and ALA groups. Additionally, the expression of mitochondrial fusion-related proteins increased. Compared with those in the STZ group, the levels of MFN2, and OPA1 were significantly greater in the TBF and ALA groups (Fig. 6B). These findings indicate that TBF mitigated DPN symptoms in a rat model by modulating mitochondrial homeostasis through activation of the AMPK signaling pathway, consistent with the results of cell experiments.

### TBF protects SCs against HG-induced cytotoxicity

SCs are considered one of the possible causes of nerve injury repair, and HG is known to be the most important feature of diabetes. The American Diabetes Association defined the average concentration of FBS as being less than 5.6 mM^19^. To simulate an uncontrolled diabetes mellitus state, we utilized 30 mM glucose culture medium to detect the impact of HG on the viability of SCs. In addition, we also used 5.6 mM glucose culture medium as a reference for normal physiological glucose levels. The SCs were cultured under HG conditions for 24, 48, and 72 h, and the cytotoxic effects of HG were evaluated using the CCK-8 assay. Considering the osmotic effect caused by HG, we used a combination of 5.6 mM NG and 24.4 mM mannitol as an osmotic control to culture the cells for 24, 48, and 72 h.

**Fig. 7:**
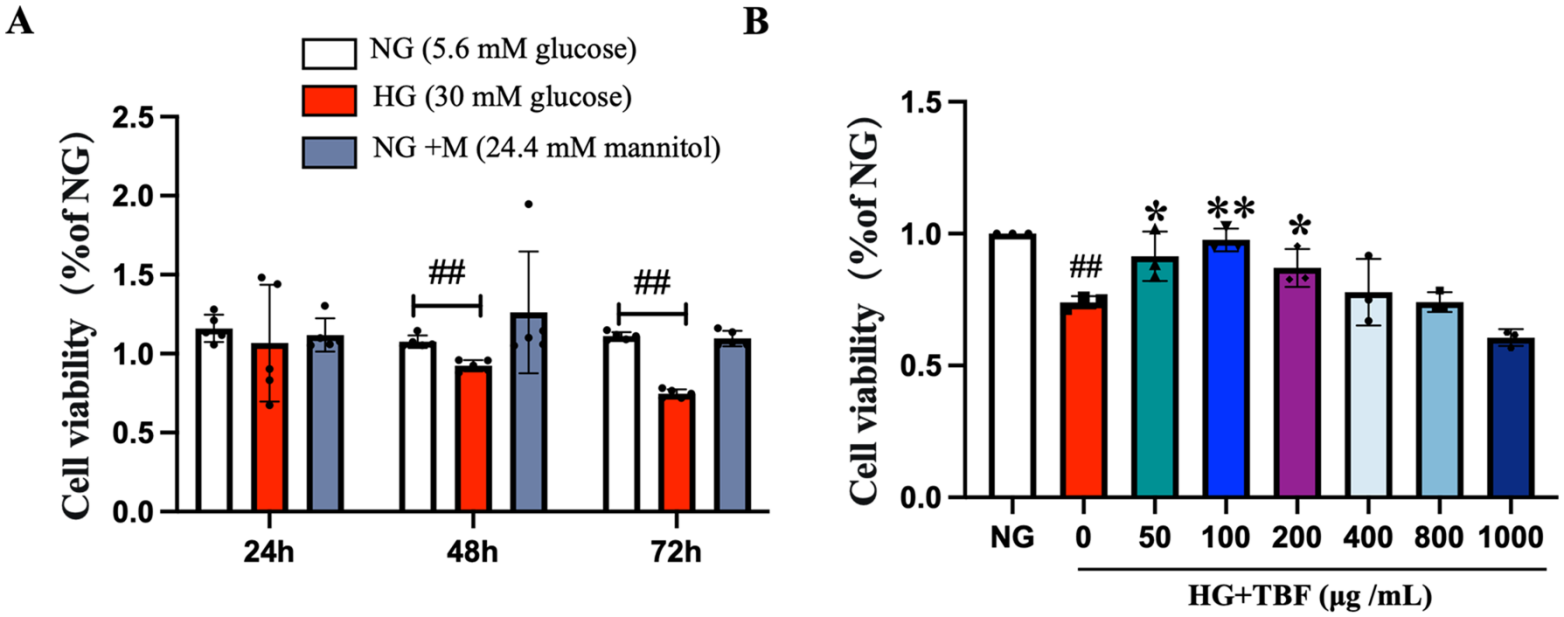
The effects of HG and TBF on the survival rate of SCs were investigated. (**A**) SCs were cultured in HG medium for 24, 48, and 72 h. An osmotic control was also included, consisting of cells incubated in 5.6 mM NG + 24.4 mM mannitol for 24, 48, and 72 h. (**B**) The SCs were subjected to HG medium with varying concentrations (50, 100, 200, 400, 800, 1000 μg/mL) of TBF for a duration of 48 h to assess the impact of TBF on cell viability. N = 3 in each group. # *p* < 0.05 and ##* p* < 0.01 versus. NG; * *p* < 0.05 and **p < 0.01 versus. HG.

The results revealed a substantial reduction in cell viability after 48 and 72 h of cultivation under HG conditions compared to that in the NG group (Fig. 7A, p < 0.01). However, no significant impact was observed within 24 h. Cells were treated with varying concentrations of TBF under HG conditions (50–1000 μg/mL) for 48 h, resulting in an increase in cell viability. This suggested that TBF restored the decreased cellular viability caused by HG. Moreover, at concentrations ranging from 50 to 800 μg/mL, TBF effectively ameliorated HG-induced cell death. Based on this, we chose 50, 100, and 200 μg/mL as the concentrations of TBF used in the present study (Fig. 7B).

### TBF protects SCs by reversing HG-induced oxidative injury, decreasing the MMP and increasing apoptosis

**Fig. 8:**
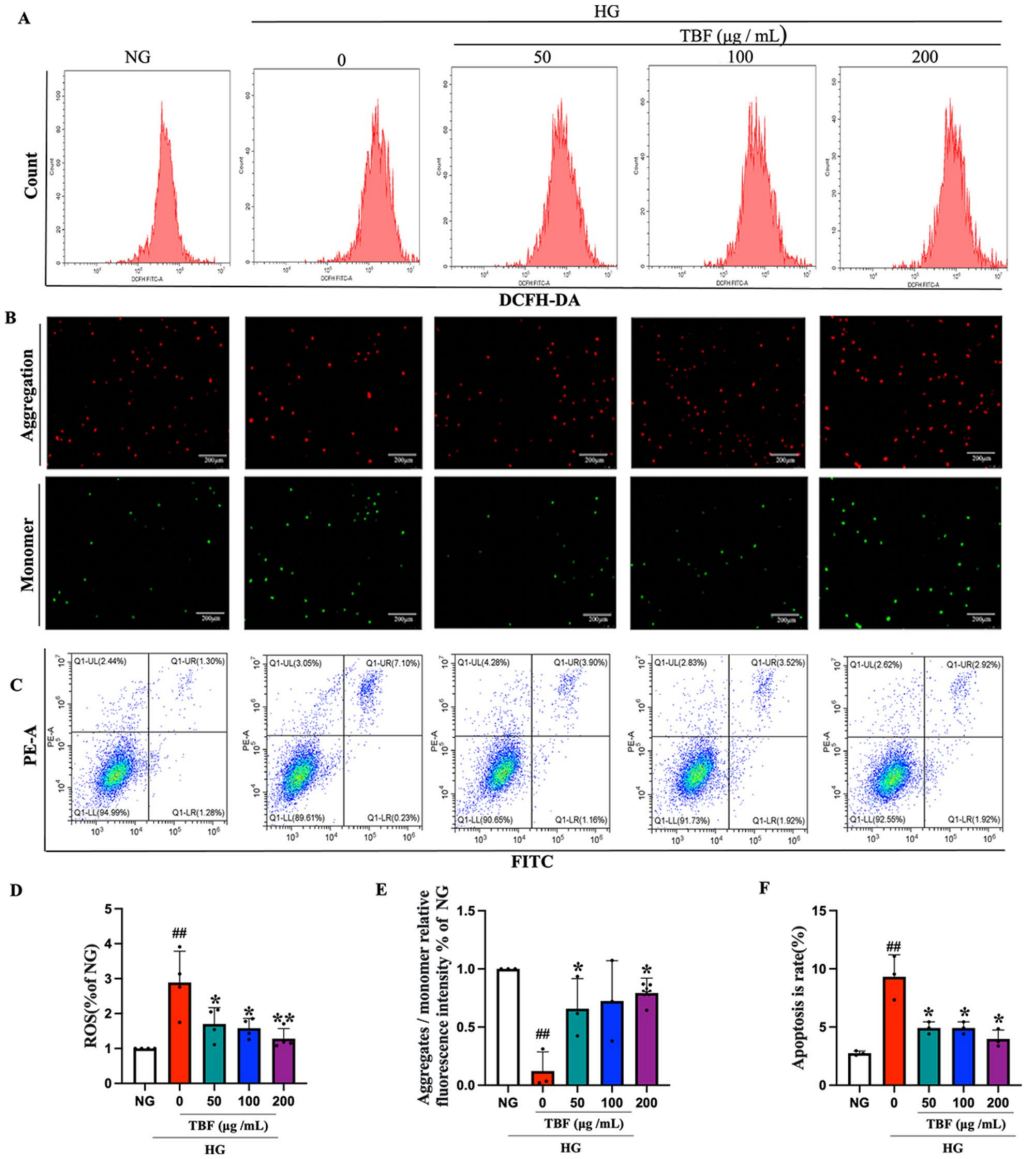
The impact of TBF on ROS production, MMP, and apoptosis in SCs. (**A**) The levels of ROS after 48 h were assayed by a flow cytometer. (**B**) The MMP at 48 h was measured using the JC-1 probe. The reduction of MMP is demonstrated by a decline in red fluorescence and a rise in green fluorescence. Bar = 200 μm. (**C**) The results of the apoptosis assay after 48 h. (**D-F**) Quantitative analysis of ROS, MMP, and apoptosis. N = 3 in each group #*p* < 0.05; ##*p* < 0.01, versus. NG. *p < 0.05; ** p < 0.01 versus. HG.

During the hyperglycemic response in diabetes mellitus, the generated ROS can have toxic effects on cells. To explore the potential protective effects of TBF on HG-induced oxidative toxicity, we utilized DCFH2-DA staining to quantify the accumulation of ROS. Flow cytometry analysis revealed a notable increase in the fluorescence intensity of DCFH-DA in SCs treated with HG for 48 h compared to that in the NG group. However, TBF (50, 100, or 200 μg/mL) strongly suppressed this increase (Fig. 8A, D). The MMP is a universal, selective indicator of mitochondrial function. The increased generation of ROS can further damage the mitochondria, resulting in a reduced MMP. The transition from red (aggregation) to green fluorescence (monomer) indicated a decrease in the MMP according to JC-1 staining. As depicted in Fig. 4B, a notable increase in red fluorescence was observed in the NG group. The MMP in the HG group rapidly decreased, accompanied by a decrease in the ratio of red fluorescence to green fluorescence, indicating that HG caused mitochondrial damage. Nevertheless, subsequent pretreatment with TBF (50 or 200 μg/mL) reversed this effect, leading to the stabilization of the MMP (Fig. 8B, E). A distinct decrease in the MMP and excessive generation of ROS often promote cell apoptosis. SC apoptosis is crucial in the pathogenesis of DPN^20,21^. Subsequently, we investigated the effects of TBF on glucose-induced apoptosis in SCs under HG conditions. The results suggested that after exposure to HG for 48 h, the level of cell apoptosis significantly increased. However, TBF pretreatment reduced the level of cell apoptosis (Fig. 8C, F). These findings suggested that TBF plays a key role in inhibiting oxidative injury, mitochondrial function injury, and cell apoptosis.

### Effects of TBF on the AMPK pathway and mitochondrial dynamics in HG-induced SCs

**Fig. 9:**
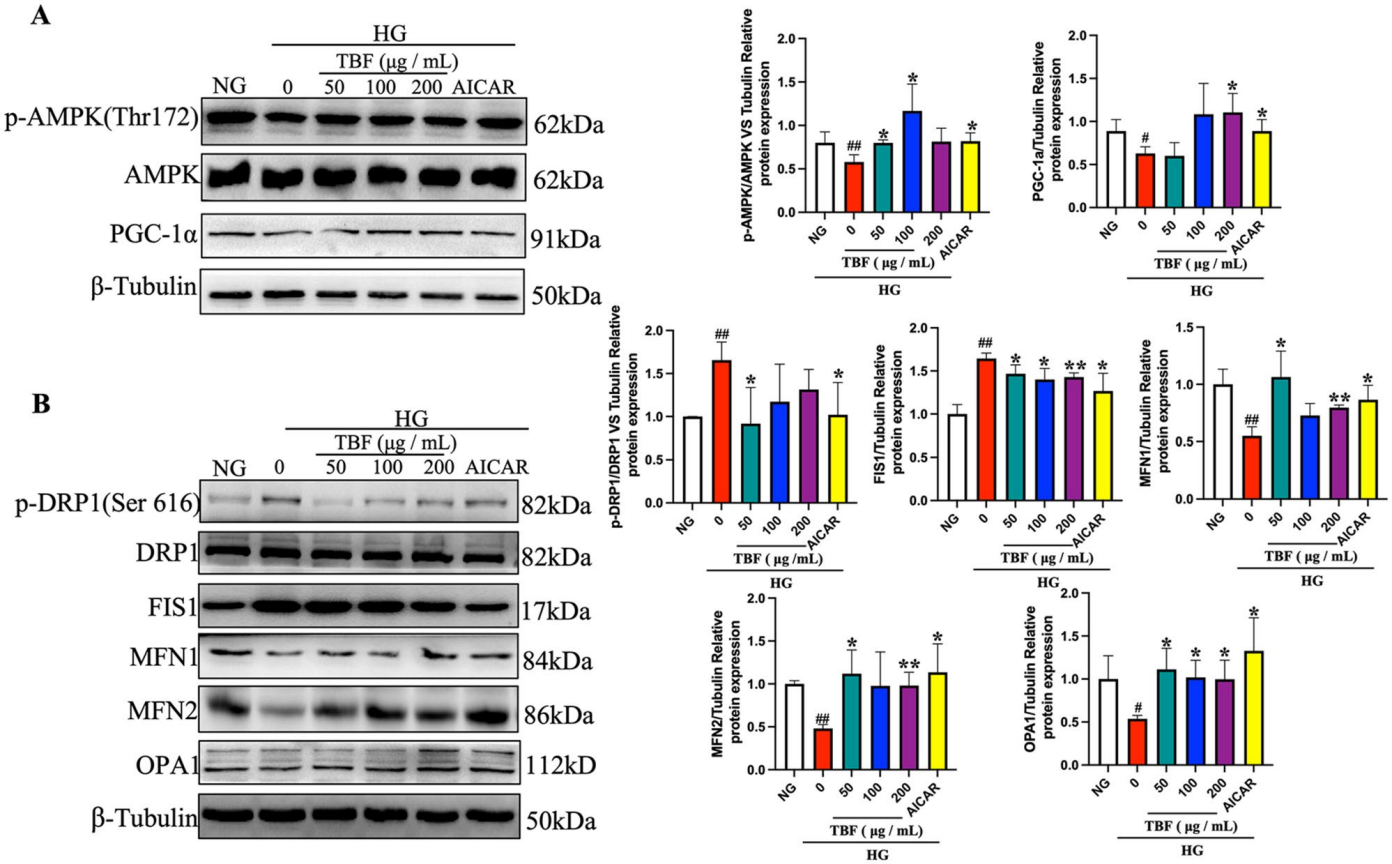
TBF regulated the AMPK pathway and the changes in its downstream mitochondrial dynamics of HG-induced SCs. (**A**) The protein levels of p-AMPK, AMPK, PGC-1α in the SCS from different groups of NG group, HG group, TBF group (HG + 50, 100, 200 μg/mL TBF), AICAR group (HG + 0.5 mM AICAR). (**B**) The protein levels of p-DRP1, DRP1, FIS1, MFN1, MFN2 and OPA1 in the SCS from different groups of NG group, HG group, TBF group (HG + 50, 100, 200 μg/mL TBF), AICAR group (HG + 0.5 mM AICAR). Data are shown as mean ± standard deviation from three independent experiments. The outcomes were normalized to the values of the NG. β-tubulin images are reused. #*p* < 0.05; ##*p* < 0.01versus. NG. *p < 0.05; ** p < 0.01 versus. HG.

Next, we validated the cellular results in SCs induced by HG. As shown in Fig. 9A, exposure of SCs to HG notably reduced the levels of p-AMPK (Thr172)/AMPK and PGC-1α. Compared to that in the HG group, the expression of p-AMPK (Thr172)/AMPK and PGC-1α was significantly greater in the TBF group than in the SC group; moreover, long-term AIACR treatment reversed this phenomenon. These findings suggested that TBF activated the AMPK pathway in HG-induced SCs. Additionally, the stimulation of the AMPK pathway by TBF was comparable to that of AICAR. Subsequently, we observed a marked decrease in the protein expression levels of MFN1, MFN2, and OPA1 in SCs exposed to HG. Additionally, a substantial increase in the expression of p-DRP1 (Ser616)/DRP1 as well as FIS1 was found in the HG group compared to the NG group (Fig. 9A). Compared with those in the HG group, the protein expression levels of p-DRP1 (Ser616)/DRP1 and FIS1 were notably decreased in the TBF and AICAR groups, while the levels of MFN1, MFN2, and OPA1 were upregulated (Fig. 9B).

## Discussion

DPN is a length-dependent peripheral neuropathy characterized by the classic "stocking-and-glove" sensory distribution pattern^22^. Its pathological hallmarks include segmental demyelination of myelinated axons, axonal degeneration, and atrophy of unmyelinated fibers, collectively leading to reduced NCV and sensory dysfunction^22^. Chronic hyperglycemia serves as the primary etiological driver of DPN^23^, impairing glucose utilization in vascular endothelial cells, SCs, and neurons—rendering these cell types particularly susceptible to glucotoxicity^24^. Current clinical medications for DPN, including neurotrophic agents (e.g., methylcobalamin, alpha-lipoic acid) and analgesics (e.g., pregabalin, gabapentin), are associated with significant side effects. Notably, DPN remains an incurable disease to date. TCM is renowned for its favorable efficacy and minimal side effects. Derived from the modified traditional classic formula Huangqi Guizhi Wuwu Decoction, TBF has been clinically demonstrated to significantly improve pain symptoms and nerve conduction velocity in DPN patients, showing promising clinical efficacy. However, the material basis and underlying mechanisms of TBF’s action remain undefined.

In this study, serum pharmacochemistry analysis via UHPLC/QTOF-MS identified 11 prototype components absorbed into the bloodstream, including nicotinamide, flavonoids, monoglycosides, aromatic compounds, phenylpropanoids, ferulic acid lactones, and benzofurans. These bioactive constituents are pharmacologically associated with anti-inflammatory, blood circulation-improving, analgesic, and antioxidant properties^25,26^, potentially contributing to TBF’s neuroprotective effects through multi-target engagement. Oxidative stress, as a core link in the occurrence and progression of DPN, can promote disease progression by exacerbating mitochondrial dysfunction. The AMPK pathway is closely associated with mitochondrial energy metabolism, and its activity is reduced in DPN^8,9^. Network pharmacology analysis has identified the AMPK signaling pathway and mitochondrial function as central nodes mediating the anti-DPN effects of TBF. Pharmacological activation of AMPK via agents like metformin—widely used in diabetes management—has demonstrated efficacy in ameliorating nerve injury and neuropathic pain through promoting myelination^27,28^.Therefore, regulating the AMPK signaling pathway may be a potential therapeutic target for DPN. The results of this study revealed that the activity of AMPK in DPN rats and cell models decreased, which was consistent with the previously reported results. In addition, In our study, streptozotocin-induced DPN rats exhibited tactile allodynia, reduced sensory/motor NCV, and SN hypoperfusion. Treatment with TBF ameliorated neuropathic pain, restored NCV parameters, and improved SN microvascular perfusion. Histopathological analysis revealed TBF-mediated preservation of myelinated fiber architecture, attenuation of axonal atrophy/swelling, and reduction in demyelination—further confirming its protective effect against nerve injury.

PGC-1α, a transcriptional co-activator downstream of AMPK, is recognized as a major regulator of mitochondrial activity in numerous tissues. Positively regulated by upstream AMPK, its downregulation leads to mitochondrial dysfunction, which promotes apoptosis of sensory neurons/Schwann cells, distal axonal degeneration, and demyelination during the progression of DPN^29^. AMPK activation under energy stress phosphorylates PGC-1α, in hyperglycemic conditions, reduced AMPK phosphorylation observed in both DPN rat sciatic nerves and HG-treated SCs correlates with mitochondrial dysfunction, aligning with literature demonstrating PGC-1α-dependent suppression of mitochondrial degeneration via NRF2 activation^30^. Strikingly, our high-dose TBF group preserved PGC-1α expression despite AMPK inhibition, suggesting compensatory mechanisms may bypass canonical AMPK regulation to sustain MFN2 upregulation and mitochondrial fusion^31^.

In addition, mitochondrial fusion and fission dynamics are central to ROS production, apoptosis pathways, and inflammatory responses. In DRG neurons exposed to high levels of glucose, mitochondrial fragmentation increases, Drp1 expression increases, and oxidative stress is positively correlated^32^. Furthermore, high glucose can stimulate an increase in the level of the Drp1/Bax complex, leading to apoptosis^33^. Increased mitochondrial fission can activate mitochondrial ROS production and affect the expression of proinflammatory mediators through the activation of NF-κB and MAPK^34^. AMPK is involved in the molecular mechanisms of aging, obesity, and protection against drug-induced liver injury by regulating the mitochondrial fusion-related protein OPA1 and mitochondrial activity and dynamics^35,36^. Recent studies have reported that the AMPK pathway has beneficial effects on diabetes, insulin resistance, diabetic myocardial microvascular injury, and diabetic nephropathy by increasing mitochondrial fusion and reducing mitochondrial fission^37–40^.This finding converges with evidence that PGC-1α directly promotes MFN2 transcription to enhance mitochondrial fusion^31^, counteracting DRP1-mediated fission. DRP1 translocation to mitochondria, driven by CDK1 phosphorylation at Ser616, exacerbates fission and ROS production in hyperglycemic states^9296^. TBF-mediated suppression of DRP1 activation—likely through AMPK-dependent phosphorylation at Ser637—may disrupt this vicious cycle, reducing mitochondrial fragmentation and cytochrome c release^41^. Conversely, MFN2 deficiency impairs calcium buffering capacity and increases Bax-mediated apoptosis, underscoring its protective role in DPN^42^. Notably, the biphasic AMPK response observed in our study mirrors Okada’s 2022 report^43^, where high-dose quetiapine suppressed AMPK via 5-HT7R inverse agonism. This suggests TBF may similarly modulate receptor signaling at higher concentrations, diverting energy toward alternative survival pathways like mTOR/ERK crosstalk^44^. The resultant PGC-1α upregulation—independent of AMPK—highlights the plasticity of mitochondrial regulatory networks, which may be therapeutically leveraged to optimize DPN treatment. MFN2, a key mitochondrial outer membrane GTPase, regulates mitochondrial energy metabolism, axonal transport, and cristae organization in both neurodegenerative and metabolic disorders^45^. Conversely, DRP1 drives mitochondrial fission through FIS1-mediated translocation to the outer mitochondrial membrane^11^. Pathological DRP1 hyperactivation promotes excessive mitochondrial fragmentation, perturbing oxidative phosphorylation and exacerbating ROS production, which triggers apoptotic cascades via cytochrome c release^46^. This fission-biased phenotype, characterized by DRP1 upregulation and/or MFN2 downregulation, is a conserved feature of metabolic diseases and diabetic neuropathy^11,47,48^. CPost-translational modifications further modulate DRP1 activity: CDK1-mediated phosphorylation at Ser616 enhances mitochondrial recruitment, whereas phosphorylation at Ser637 by AMPK suppresses fission^49,50^. Hyperglycemic stress disrupts this phosphorylation balance, favoring DRP1 activation and ROS overproduction—a vicious cycle linked to neuropathic pain pathogenesis. Indeed, DRP1 inhibition with mdivi-1 preserves mitochondrial integrity in lipotoxicity models by restoring Bcl-2/Bax ratios and suppressing NLRP3 inflammasome activation^50^. In our study, hyperglycemic insult reduced MFN2 and OPA1 levels in SCs and the substantia nigra of STZ-induced DPN rats, concurrent with increased p-DRP1/DRP1 ratios. These findings align with OPA1’s role in maintaining crista structure, mtDNA stability, and redox homeostasis. TBF treatment restored MFN2/OPA1 expression and normalized DRP1 phosphorylation, highlighting its potential to rebalance mitochondrial dynamics and mitigate oxidative stress in DPN.

However, this study is limited to specific animal models and cell lines, which may not fully recapitulate the complex pathophysiology of human DPN. Additionally, the efficacy of TC M compound administration is influenced by synergistic/antagonistic interactions among components and in vivo pharmacokinetic processes, yet the interactive mechanisms of multi-components remain uncharacterized. The biphasic AMPK response induced by high-dose TBF lacks a defined therapeutic window. Key targets predicted by network pharmacology (e.g., HSP90AA1, EGFR) have not been systematically validated, and these unvalidated targets may synergize with the AMPK pathway via processes such as oxidative stress regulation, apoptosis inhibition, and proteostasis maintenance. Non-hematological pathways like intestinal flora regulation by TCM compounds may also play critical roles in DPN.

To address these limitations, future studies will: 1) utilize AMPK gene-knockout models combined with omics technologies to dissect the regulatory mechanism of the AMPK/PGC-1α/MFN2 axis; 2) construct component-target interaction maps via molecular docking and surface plasmon resonance (SPR); 3) define the therapeutic window through dose–response analyses; 4) validate key target binding activities via high-throughput screening and map crosstalk between AMPK and other pathways (e.g., mTOR/NF-κB); and 5) explore non-hematological mechanisms like intestinal flora regulation using 16S rRNA sequencing and fecal microbiota transplantation. These approaches will comprehensively reveal the multi-target synergism and trans-system regulatory patterns of TBF, facilitating the clinical translation of precision TCM therapy for DPN.

## Conclusions

**Fig. 10:**
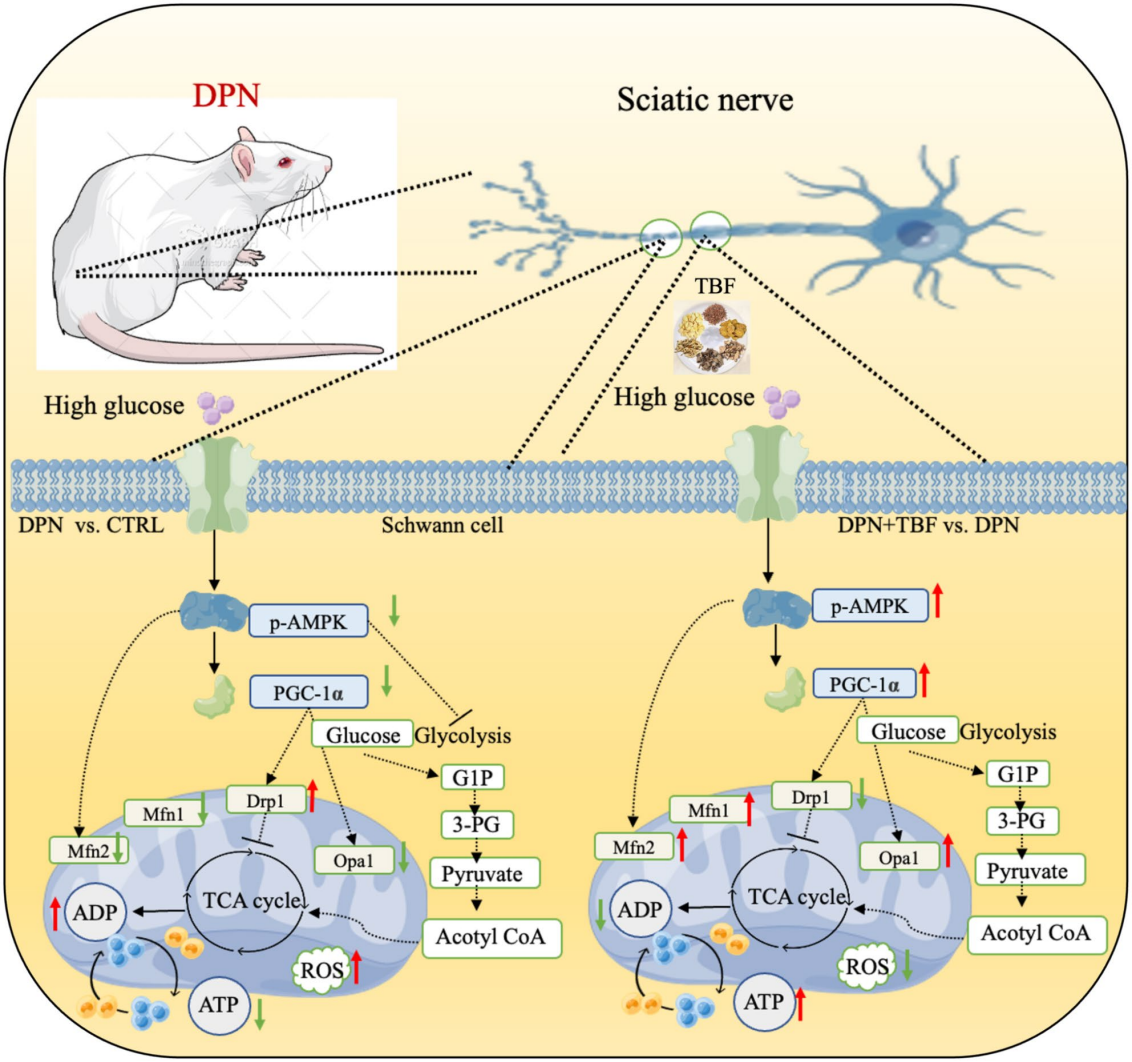
Diagram of the mechanism of TBF in the treatment of DPN.

Our study shows that TBF exerts an effective protective effect on nerve damage by activating AMPK-PGC-1α-MFN2 pathway, stabilizing mitochondrial division and fusion, and inhibiting Schwann cell apoptosis and oxidative stress (Fig. 10). Our results provide a new perspective on the mechanism of anti-neural damage of TBF and suggest it as a promising strategy for the treatment of DPN. However, the ingredients of Chinese medicine compound are complicated. Further research is needed to prioritize identifying these components to better understand their mechanisms and potential therapeutic applications.

## Supplementary Information

Supplementary Information 1.

Supplementary Information 2.

Supplementary Table 1.

Supplementary Table 2.

Supplementary Table 3 .

Supplementary Table 4.

## Data Availability

The data that support the findings of this study are available from the corresponding author upon reasonable request.

## Abbreviations

DPN: Diabetic peripheral neuropathy
TCM: Traditional Chinese medicine
TBF: Tang Bi formula
UHPLC/QTOF-MS: Ultra-performance liquid chromatography-quadrupole time-of-flight mass spectrometry
ALA: Alpha-lipoic acid
NCV: Nerve conduction velocity
HG: High glucose
SC: Schwann cell
STZ: Streptozotocin
SN: Sciatic nerve
MS: Mass spectrometry
BP: Biological process
CC: Cellular component
MF: Molecular function
KEGG: Kyoto Encyclopedia of Genes and Genomes
SD: Sprague–Dawley
PWT: Paw withdrawal threshold
MWT: Mechanical hyperalgesia assessment
SNCV: Sensory nerve conduction velocity
MNCV: Motor nerve conduction velocity
DRP1: Dynamin-related protein 1
FIS1: Mitochondrial fission protein 1
MNF1: Mitochondrial fusion protein 1
OPA1: Optic atrophy protein 1
PGC-1α: Peroxisome proliferator-activated receptor-γ coactivator 1α
MNF2: Mitochondrial fusion protein 2
