# Supplementary figures and images for: Tang Bi formula alleviates diabetic sciatic neuropathy via AMPK/PGC-1α/MFN2 pathway activation
Source: Sci Rep. 2025 Jul 11;15:25069. doi: 10.1038/s41598-025-10513-0 (PMC12254306; doi:10.1038/s41598-025-10513-0)

**Supplementary Information File-Western blo**t

**Fig 6**


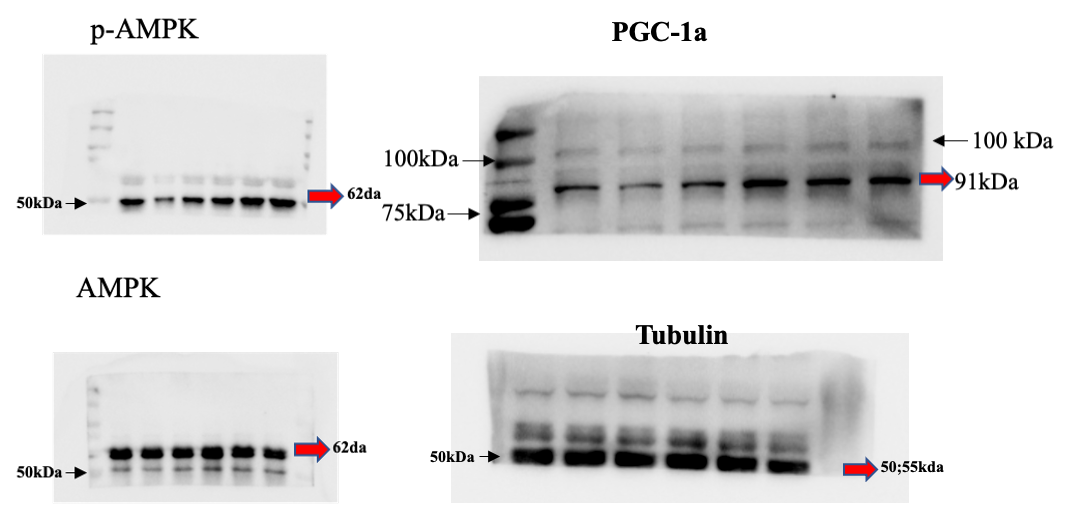


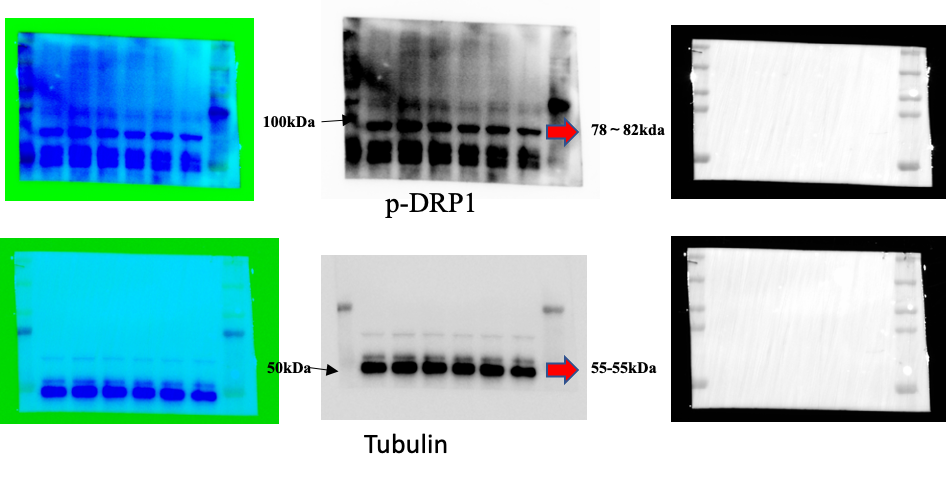


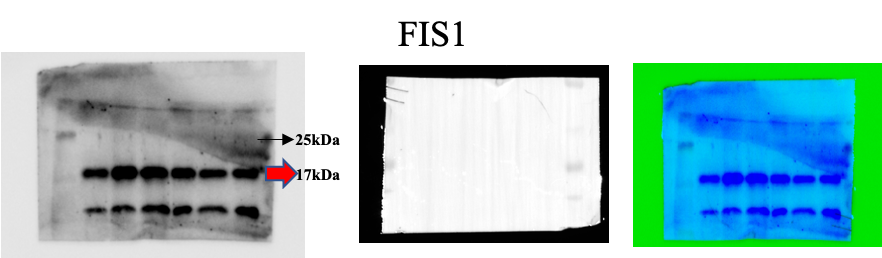


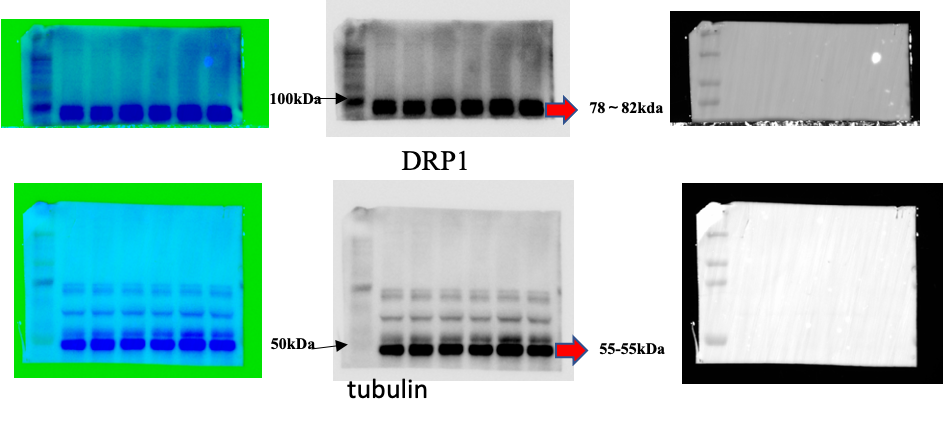


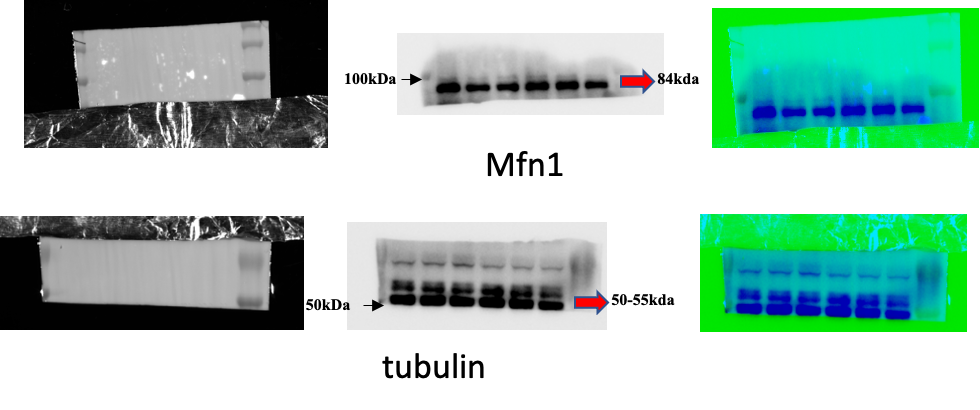


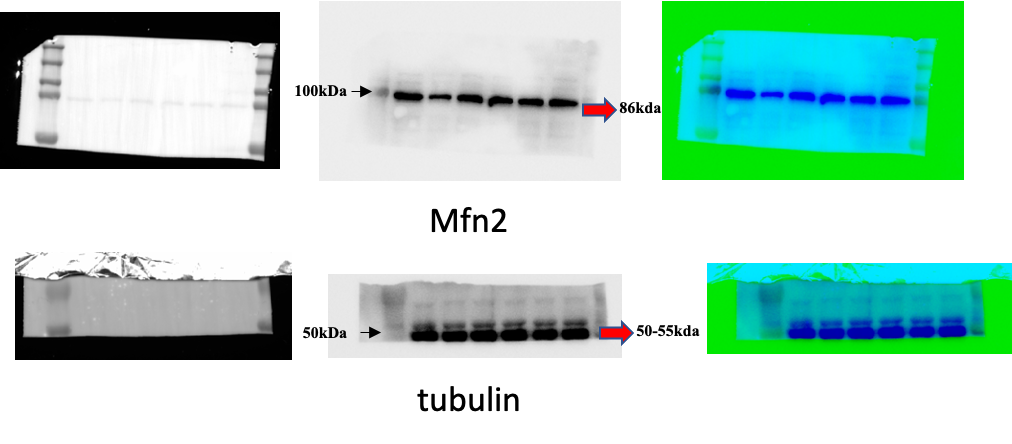


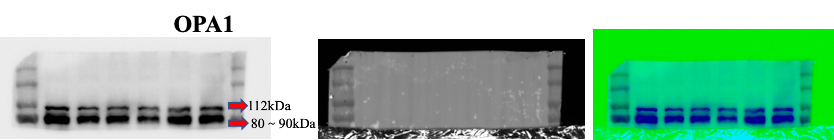


**Fig 9**


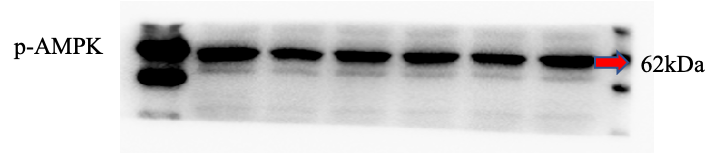


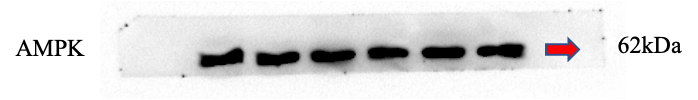


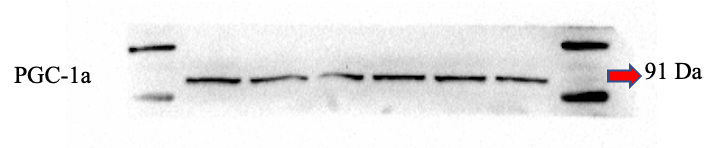


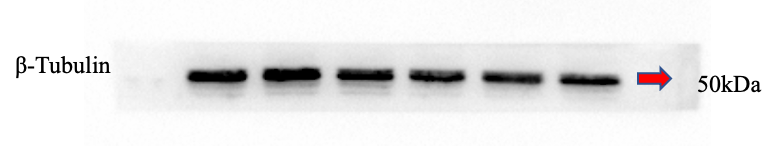


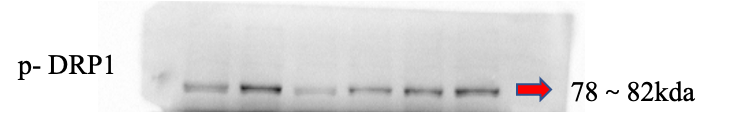


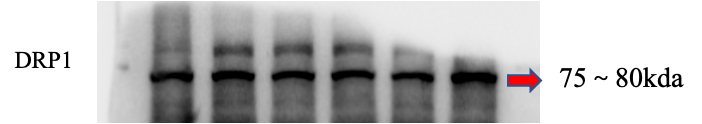


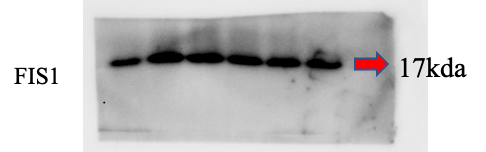


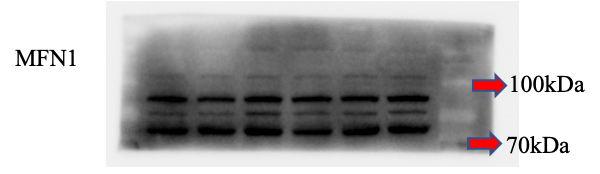


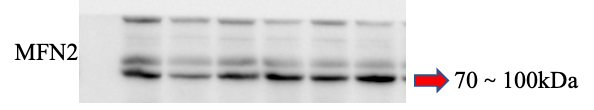


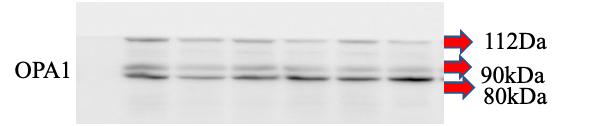

Supplement: Supplementary file 1 — Supplementary Information 1. [file 41598_2025_10513_MOESM1_ESM.docx]
