# Supplementary material for: Tang Bi formula alleviates diabetic sciatic neuropathy via AMPK/PGC-1α/MFN2 pathway activation
Source: Sci Rep. 2025 Jul 11;15:25069. doi: 10.1038/s41598-025-10513-0 (PMC12254306; doi:10.1038/s41598-025-10513-0)
Supplement: Supplementary file 2 — Supplementary Information 2. [file 41598_2025_10513_MOESM2_ESM.docx]

**Supplementary Section**

**
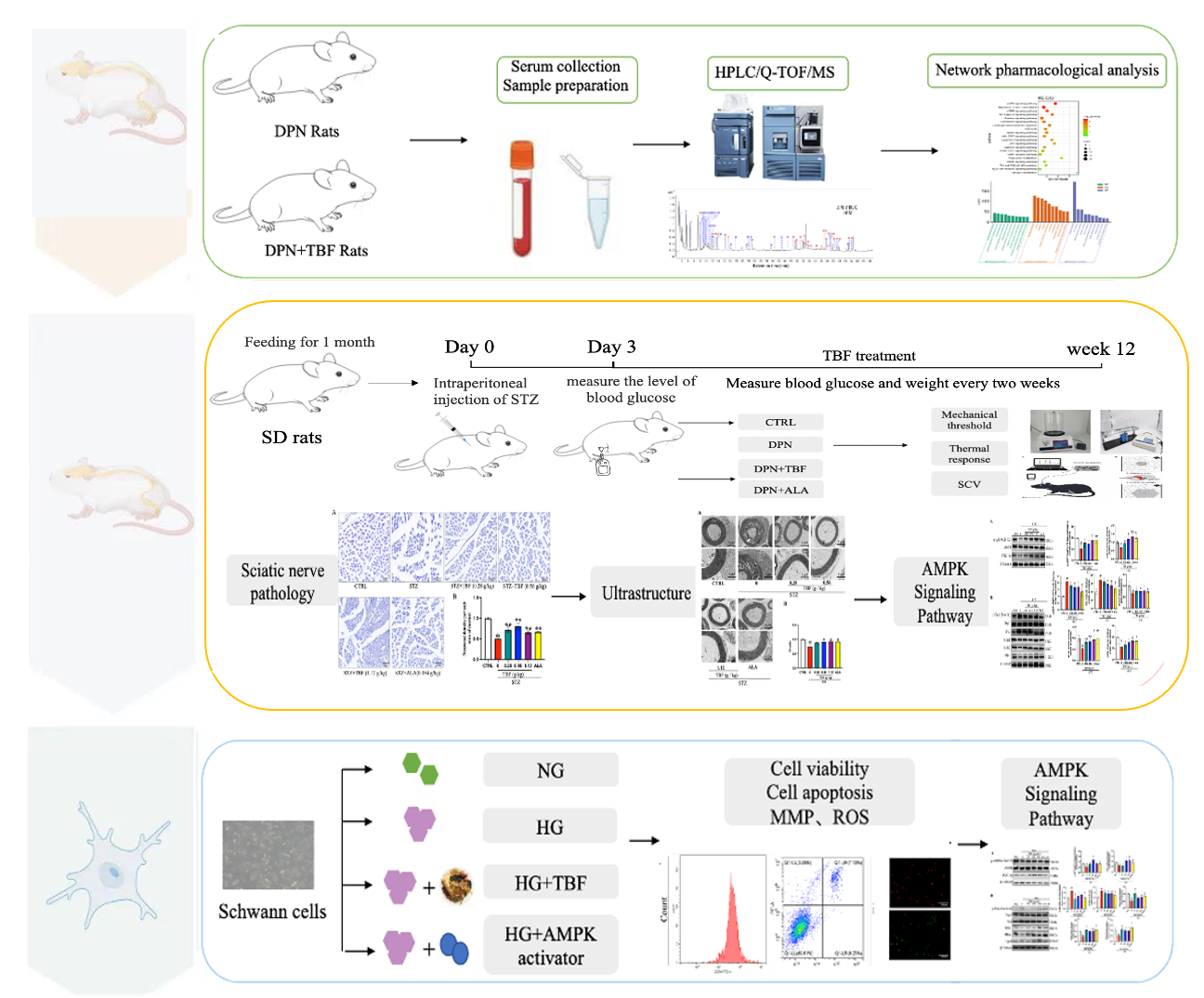
**

**Supplementary figure 1.** Schematic overview of the experimental design and proposed mechanism of Tang Bi formula in alleviating diabetic sciatic neuropathy.
