# Supplementary material for: Tang Bi formula alleviates diabetic sciatic neuropathy via AMPK/PGC-1α/MFN2 pathway activation
Source: Sci Rep. 2025 Jul 11;15:25069. doi: 10.1038/s41598-025-10513-0 (PMC12254306; doi:10.1038/s41598-025-10513-0)
Supplement: Supplementary file 3 — Supplementary Table 1. [file 41598_2025_10513_MOESM3_ESM.docx]

**Table 1 Components of the TBF.**

| **English name** | **Chinese name** | **Latin name** | **Family** | **Product batch number** | **Weight (g)** | **Part used** |
| --- | --- | --- | --- | --- | --- | --- |
| Angelica | Danggui | Angelica sinensis | Umbelliferae | 220860421 | 9 | Root |
| Astragalus | Huangqi | Astragalus membranaceus | Fabaceae | 221061041 | 15 | Root |
| Notoginseng | Sanqi | Panax notoginseng | Araliaceae | 73011211 | 3 | Root |
| Sappanwood | Sumu | Caesalpinia sappan | Fabaceae | 220761001 | 9 | lumber |
| Cinnamon Twig | Guizhi | Cinnamomum cassia | Lauraceae | 220661101 | 9 | Twig and Bark |
| Chuanxiong | Chuanxiong | Ligusticum chuanxiong | Umbelliferae | 221260231 | 15 | Rhizome |
| White Peony Root | Baishao | Paeonia lactiflora | Ranunculaceae | 220960581 | 9 | Root |
| Aconite | Fuzi | Aconitum carmichaelii | Aconitum | 72062411 | 3 | Rhizome |
| Mulberry Twig | Sangzhi | Morus alba | Moraceae | 22096046 | 15 | Twig and Bark |
