# Supplementary material for: Tang Bi formula alleviates diabetic sciatic neuropathy via AMPK/PGC-1α/MFN2 pathway activation
Source: Sci Rep. 2025 Jul 11;15:25069. doi: 10.1038/s41598-025-10513-0 (PMC12254306; doi:10.1038/s41598-025-10513-0)
Supplement: Supplementary file 4 — Supplementary Table 2. [file 41598_2025_10513_MOESM4_ESM.docx]

Table2 Standard information sheet

| **IDX** | **Name** | **Molecular formula** | **Precise mass number** | **CAS number** | **Art.No.** |
| --- | --- | --- | --- | --- | --- |
| 1 | Cinnamic acid | C_9_H_8_O_2_ | 148.0524 | 621-82-9 | – |
| 2 | Z-Ligustilide | C_12_H_14_O_2_ | 190.0994 | 81944-09-4 | – |
| 3 | Ferulic acid | C_10_H_10_O_4_ | 194.0579 | 1135-24-6 | – |
| 4 | Senkyunolide I | C_12_H_16_O_4_ | 224.1049 | 94596-28-8 | ST13270120 |
| 5 | Formononetin | C_16_H_12_O_4_ | 268.0736 | 485-72-3 | – |
| 6 | Calycosin | C_16_H_12_O_5_ | 284.0685 | 20575-57-9 | – |
| 7 | Brazilin | C_16_H_14_O_5_ | 286.0841 | 474-07-7 | ST07800120 |
| 8 | L-Epicatechin | C_15_H_14_O_6_ | 290.0790 | 490-46-0 | – |
| 9 | Protosappanin | C_16_H_16_O_6_ | 304.0947 | 102036-29-3 | ST07780120 |
| 10 | Astraisoflavan-7--O--β-D-glucoside | C_23_H_28_O_10_ | 464.1682 | 136087-29-1 | – |
| 11 | Paeoniflorin | C_23_H_28_O_11_ | 480.0632 | 23180-57-6 | – |
| 12 | Oxypaeoniflorin | C_23_H_28_O_12_ | 496.1581 | 39011-91-1 | – |
| 13 | Deacetylhypaconitine | C_31_H_43_NO_9_ | 573.2938 | 63238-66-4 | – |
| 14 | Benzoylmesaconine | C_31_H_43_NO_10_ | 589.2887 | 63238-67-5 | – |
| 15 | Complanatuside | C_28_H_32_O_16_ | 624.1690 | 116183-66-5 | – |
| 16 | Astragaloside III | C_41_H_68_O_14_ | 784.4609 | 84687-42-3 | – |
| 17 | Astragaloside II | C_43_H_70_O_15_ | 826.4715 | 84676-89-1 | – |
| 18 | Notoginsenoside R1 | C_47_H_80_O_18_ | 933.5345 | 80418-24-2 | – |
| 19 | Ginsenoside Re | C_48_H_82_O_18_ | 946.5501 | 51542-56-4 | – |
| 20 | Mulberroside F | C_26_H_30_O_14_ | 566.1636 | 193483-95-3 | – |
| 21 | Kuwanon A | C_25_H_24_O_6_ | 420.1573 | 62949-77-3 | – |
| 22 | Ginsenoside Rb3 | C_53_H_90_O_22_ | 1078.5924 | 68406-26-8 | – |
| 23 | Cinnamyl alcohol | C_9_H_10_O | 134.0732 | 104-54-1 | ST06990120 |
| 24 | Protocatechuic acid | C_7_H_6_O_4_ | 154.0266 | 99-50-3 | – |
| 25 | 2-Methoxycinnamic acid | C_10_H_10_O_3_ | 178.0630 | 6099-03-2 | ST04260120 |
| 26 | Senkyunolide A | C_12_H_16_O_2_ | 192.1150 | 63038-10-8 | ST10580120 |
| 27 | Senkyunolide H | C_12_H_16_O_4_ | 224.1049 | 94596-27-7 | ST13470105 |
| 28 | Isoliquiritigenin | C_15_H_12_O_4_ | 256.0736 | 961-29-5 | ST09620120 |
| 29 | Oroxylin A | C_16_H_12_O_5_ | 284.0685 | 480-11-5 | – |
| 30 | Biochanin A | C_16_H_12_O_5_ | 284.0685 | 491-80-5 | ST03270120 |
| 31 | Sappanchalcone | C_16_H_14_O_5_ | 286.0841 | 94344-54-4 | DS0367-0005 |
| 32 | Hematoxylin | C_16_H_14_O_6_ | 302.0790 | 517-28-2 | DS0181-0020 |
| 33 | Astragalin | C_21_H_20_O_11_ | 448.1006 | 480-10-4 | – |
| 34 | Alibiflorin | C_23_H_28_O_11_ | 480.1632 | 39011-90-0 | – |
| 35 | MulberrosideA | C_26_H_32_O_14_ | 568.1792 | 102841-42-9 | ST05680120 |
| 36 | Benzoylpaeoniflorin | C_30_H_32_O_12_ | 584.1894 | 38642-49-8 | DB0053-0020 |
| 37 | benzoylaconine | C_32_H_45_NO_10_ | 603.3043 | 466-24-0 | – |
| 38 | Astragaloside | C_41_H_68_O_14_ | 784.4609 | 83207-58-3 | – |
| 39 | Ginsenoside Rg1 | C42H72O14 | 800.4922 | 22427-39-0 | – |
| 40 | Isoastragaloside I | C45H72O16 | 868.4820 | 84676-88-0 | – |
| 41 | Ginsenoside Rd | C_48_H_82_O_18_ | 946.5501 | 52705-93-8 | ST05970120 |
| 42 | Kuwanon H | C_45_H_44_O_11_ | 760.2884 | 76472-87-2 | – |
| 43 | Mulberroside C | C_24_H_26_O_9_ | 458.1577 | 102841-43-0 | – |
| 44 | Ginsenoside Rc | C53H90O22 | 1078.5924 | 11021-14-0 | – |
| 45 | Ginsenoside Rb1 | C54H92O23 | 1108.6029 | 41753-43-9 | – |
