# Supplementary material for: Tang Bi formula alleviates diabetic sciatic neuropathy via AMPK/PGC-1α/MFN2 pathway activation
Source: Sci Rep. 2025 Jul 11;15:25069. doi: 10.1038/s41598-025-10513-0 (PMC12254306; doi:10.1038/s41598-025-10513-0)
Supplement: Supplementary file 5 — Supplementary Table 3 . [file 41598_2025_10513_MOESM5_ESM.docx]

**List of chemical constituents identified by in vitro liquid mass analysis of TBF**

| **Number** | **Retention time（min）** | **Molecular formula** | **Measured mass-charge ratio *m/z*** | **Mass error**  **(ppm)** | **Secondary mass spectrometry data** | **Name of compound** | **Compound source** |
| --- | --- | --- | --- | --- | --- | --- | --- |
| 1 | 1.65 | C_6_H_6_N_2_O | 123.0556 | 2.46 | 267.0642,239.0700,213.0545,203.0698,185.0593,165.0694,129.0690,73.0281 | Nicotinamide | Chuanxiong |
| 2 | 3.24 | C_5_H_5_N_5_ | 136.0620 | 1.48 | 267.0640,239.0695,221.0589,213.0543,185.0590,175.0744,157.0642,129.0692,73.0280,55.0177 | Adenine | Sanqi |
| 3 | 4.61 | C_9_H_8_O_2_ | 166.0865 | 2.03 | 267.0640,239.0693,221.0590,213.0545,203.0699,185.0590,175.0750,157.0643,129.0692,115.0535,73.0281,55.0177 | 3-phenyl-2-acrylic acid | Chuanxiong |
| 4^*^ | 5.41 | C_7_H_6_O_4_ | 153.0197 | 2.60 | 229.0510,163.0407,135.0457,121.0301,109.0299,91.0560 | Protocatechuic acid | Guizhi |
| 5^*^ | 7.29 | C_16_H_14_O_6_ | 301.0727 | 3.31 | 267.0640,239.0696,221.0590,213.544,203.0697,185.0593,157.0644,129.0692,73.0281,55.0179 | Hematoxylin | Sumu |
| 6 | 8.00 | C_24_H_39_NO_9_ | 486.2715 | 3.50 | 438.2492,406.2220,356.1832,169.1008,140.1065,94.0646,57.0695 | Mesaconitine | Fuzi |
| 7 | 8.42 | C_10_H_10_O_2_ | 163.0756 | 1.23 | 436.2697,404.2426,219.1157,154.1221,108.0806,58.0653 | Safrole | Chuanxiong |
| 8 | 8.88 | C_16_H_12_O_5_ | 285.0758 | 0.00 | 231.0669,230.0583,229.0511,216.0431,215.0354,213.0559,211.0407,159.0457,109.0298,73.0296 | brazilein | Sumu |
| 9^*^ | 9.03 | C_26_H_32_O_14_ | 567.1740 | 3.70 | 229.0488,213.0543,185.0589,168.0693,157.0644,131.0490,91.0541,68.9972,55.0179 | MulberrosideA | Sangzhi |
| 10^*^ | 9.07 | C_23_H_28_O_12_ | 495.1531 | 4.65 | 151.0404,137.0248,123.0458,109.0302,97.0298,83.0142,57.0355 | Oxypaeoniflorin | Baishao |
| 11 | 9.12 | C_14_H_12_O_4_ | 245.0805 | −1.64 | 241.0516 | 2,3',4,5'-four hydroxyl two styren | Sangzhi |
| 12 | 9.52 | C_8_H_6_O_3_ | 151.0392 | 1.33 | 121.0302,77.0407 | 1,4-cyclohexadiene-1,2-dicarboxylic anhydride | Danggui |
| 13 | 9.64 | C_16_H_14_O_6_ | 303.0869 | 1.99 | 151.0753,133.0649,121.0643,105.0337,97.0280,85.0285,77.0386 | Hesperidin | Huangqi |
| 14 | 9.83 | C_16_H_14_O_6_ | 303.0868 | 1.66 | 165.0553,121.0304,77.0408 | Sappanone B | Sumu |
| 15 | 10.15 | C_16_H_14_O_6_ | 303.0870 | 2.32 | 162.8400,96.9608 | Protosappanin C | Sumu |
| 16^*^ | 10.32 | C_16_H_14_O_5_ | 285.0781 | 4.54 | 89.0384,78.0461,63.0228,52.0306 | Brazil hematoxylin | Sumu |
| 17 | 10.35 | C_16_H_14_O_6_ | 303.0873 | 3.31 | 239.0695,221.0583,197.0595,165.0695,147.0433,123.0437,102.0467,68.9970,55.0180 | Sappanone B | Sumu |
| 18 | 11.10 | C_24_H_39_NO_8_ | 470.2759 | 2.13 | 221.0585.147.0432,68.9973 | Hypaconine | Fuzi |
| 19 | 11.27 | C_24_H_39_NO_7_ | 454.2809 | 1.99 | 153.0542,105.0693,91.0540,79.0544,77.0385,55.0540 | Fuziline | Fuzi |
| 20^*^ | 11.88 | C_16_H_16_O_6_ | 303.0880 | 1.98 | 129.0689,115.0525,107.0485,97.0642,91.0540,79.0542,77.0384,55.0540 | Protosappanin B | Sumu |
| 21 | 12.03 | C_16_H_14_O_5_ | 287.0920 | 2.10 | 285.0763,270.0527,137.0226 | 2,2',5'-trihydroxy-4- methoxychalcone | Huangqi |
| 22^*^ | 12.27 | C_15_H_14_O_6_ | 289.0729 | 4.15 | 133.0642,117.0694,105.0695,91.0541,77.0385,65.0385,55.0177 | Epicatechin | Guizhi |
| 23^*^ | 12.42 | C_26_H_30_O_14_ | 611.1634 | 2.62 | 284.0335,255.0305,227.0357 | Melanoside F | Sangzhi |
| 24^*^ | 13.42 | C_23_H_28_O_11_ | 525.1633 | 3.62 | 133.0641,117.0691,105.0694,91.0540,77.0385,67.0537,55.0541 | Albiflorin | Baishao |
| 25 | 13.57 | C_23_H_28_O_11_ | 481.1721 | 3.33 | 197.0806,179.0697,161.0593,133.0648,105.0335,77.0385 | Albiflorin | Baishao |
| 26^*^ | 16.06 | C_23_H_28_O_11_ | 525.1631 | 3.24 | 318.6281,175.5030,121.0303 | Paeoniflorin | Baishao |
| 27^*^ | 17.89 | C_9_H_8_O_2_ | 193.0514 | 4.14 | 269.0460,134.0380 | Cinnamic acid | Guizhi |
| 28 | 18.03 | C_10_H_8_O_3_ | 177.0547 | 0.57 | 461.1100,299.0574 | 6-methoxycoumarin | Danggui |
| 29 | 19.82 | C_16_H_12_O_5_ | 285.0771 | 4.58 | 617.3174,365.7835,248.2022,162.8372,121.0293 | Hematoxylone A | Sumu |
| 30 | 23.28 | C_16_H_12_O_5_ | 285.0761 | 1.06 | 931.5295,799.4865,637,4332,475.3790,161.0462,89.0253 | Wogonin | Huangqi |
| 31 | 24.02 | C_12_H_16_O_3_ | 209.1176 | 1.92 | 268.0383,239.0357,211.0410,184.0531,135.0093,91.0197 | Senkyunolide | Chuanxiong |
| 32 | 24.31 | C_12_H_16_O_3_ | 209.1175 | 1.44 | 324.9508,121.0304 | Senkyunolide G | Chuanxiong |
| 33 | 25.07 | C_22_H_22_O_10_ | 447.1293 | 1.57 | 799.4862,637.4335,475.3798,161.0463,89.0252 | Mullein isoflavones 7-O-glucoside | Huangqi |
| 34 | 31.44 | C_12_H_14_O_3_ | 207.1018 | 0.97 | 945.5440,799.4849,637.4319,475.3790,161.0462,89.0250 | 4-hydroxy-3-butylphthalide | Chuanxiong |
| 35^*^ | 32.44 | C_21_H_20_O_11_ | 447.0951 | 4.03 | 301.1086,286.0847,135.0451,121.0299 | Astragalin | Sangzhi |
| 36 | 34.95 | C_12_H_14_O_3_ | 207.1018 | 0.97 | 325.1092,253.0514 | 3-(4-ethylbenzoyl) propionic acid | Chuanxiong |
| 37 | 36.86 | C_23_H_28_O_11_ | 481.1711 | 1.25 | 165.0553,121.0302 | Astragaloside C | Hunagqi |
| 38^*^ | 39.27 | C_31_H_43_NO_10_ | 634.2890 | 3.31 | 119.0512,91.0198 | Benzoyl-neoaconitine | Fuzi |
| 39^*^ | 39.28 | C_16_H_14_O_5_ | 285.0781 | 4.56 | 252.0432,223.0411,195.0462,132.0226,91.0197 | Sappanchalcone | Sumu |
| 40^*^ | 39.62 | C_28_H_32_O_16_ | 669.1690 | 2.69 | 1107.5962,945.5397,783.4865,621.4319,459.3812 | Complanatuside | Hunagqi |
| 41^*^ | 41.30 | C_32_H_45_NO_10_ | 648.3042 | 2.62 | 1077.5845,945.5387,783.4857,621.4336,459.3814 | Benzoylaconitine | Fuzi |
| 42^*^ | 41.94 | C_47_H_80_O_18_ | 977.5354 | 2.76 | 1077.5860,783.4886,621.4375,459.3816 | Notoginsenoside R1 | Sanqi |
| 43^*^ | 42.35 | C_16_H_12_O_5_ | 283.0620 | 2.82 | 783.4512,651.4076,489.3582,89.0247 | Calycosin | Hunagqi |
| 44^*^ | 42.77 | C_31_H_43_NO_9_ | 618.2937 | 2.75 | 783.4552,621.3965,489.3588,161.0461,101.0242 | Deacetylhypaconitine | Fuzi |
| 45^*^ | 42.84 | C_42_H_72_O_14_ | 845.4925 | 2.48 | 945.5433,783.4889,621.4349 | Ginsenoside Rg1 | Sanqi |
| 46^*^ | 42.98 | C_48_H_82_O_18_ | 991.5504 | 2.12 | 825.4710,765.4425,161.0462,59.0143 | Ginsenoside Re | Sanqi |
| 47^*^ | 43.67 | C_23_H_28_O_10_ | 463.1624 | 3.24 | 268.0377,165.9907,110.0010 | Astragalus isoflavanside | Hunagqi |
| 48^*^ | 44.18 | C_24_H_26_O_9_ | 503.1577 | 3.58 | 268.0377,239.0353,211.0406,195.0454,132.0223 | Mulberroside C | Sangzhi |
| 49^*^ | 46.41 | C_30_H_32_O_12_ | 629.1895 | 3.02 | 129.0690,1115.0538,103.0535,91.0541,77.0385,67.0544,55.0539 | Benzoylpaeoniflorin | Baishao |
| 50^*^ | 48.65 | C_15_H_12_O_4_ | 255.0670 | 2.73 | 581.1811,539.1705,471.1437,419.1501,379.1035,353.1033,177.0921 | Isoliquiritigenin | Sumu |
| 51^*^ | 49.04 | C_16_H_12_O_4_ | 267.0674 | 4.10 | 335.0570,231.0671,175.0769,151.0041,125.0246 | Formononetin | Huangqi |
| 52^*^ | 49.58 | C_54_H_92_O_23_ | 1153.6015 | 0.35 |  | Ginsenoside Rb1 | Sanqi |
| 53^*^ | 50.09 | C_53_H_90_O_22_ | 1123.5906 | 0.00 |  | Ginsenoside Rc | Sanqi |
| 54^*^ | 50.82 | C_53_H_90_O_22_ | 1123.5924 | 1.60 |  | Ginsenoside Rb3 | Sanqi |
| 55^*^ | 50.90 | C_41_H_68_O_14_ | 829.4598 | 0.84 |  | Astragaloside | Huangqi |
| 56^*^ | 51.14 | C_41_H_68_O_14_ | 829.4598 | 0.84 |  | Astragaloside III | Huangqi |
| 57^*^ | 51.68 | C_48_H_82_O_18_ | 991.5492 | 0.91 |  | Ginsenoside Rd | Sanqi |
| 58^*^ | 52.43 | C_43_H_70_O_15_ | 871.4714 | 1.95 |  | Astragaloside II | Huangqi |
| 59^*^ | 52.70 | C_16_H_12_O_5_ | 283.0618 | 2.12 |  | Biochanin A | Sumu |
| 60^*^ | 53.31 | C_16_H_12_O_5_ | 283.0625 | 4.59 |  | Oroxylin A | Huangqi |
| 61 | 56.88 | C_12_H_14_O_2_ | 191.1069 | 1.05 |  | Z-Ligustilide | Chuanxiong |
| 62^*^ | 57.10 | C_45_H_44_O_11_ | 759.2820 | 1.19 |  | Kuwanon H | Sangzhi |
| 63^*^ | 57.73 | C_25_H_24_O_6_ | 419.1510 | 2.38 |  | Kuwanon A | Sangzhi |
