# Supplementary material for: Tang Bi formula alleviates diabetic sciatic neuropathy via AMPK/PGC-1α/MFN2 pathway activation
Source: Sci Rep. 2025 Jul 11;15:25069. doi: 10.1038/s41598-025-10513-0 (PMC12254306; doi:10.1038/s41598-025-10513-0)
Supplement: Supplementary file 6 — Supplementary Table 4. [file 41598_2025_10513_MOESM6_ESM.docx]

**Table 4 List of chemical constituents identified by in vitro liquid mass analysis of TBF** **serum sample**

| **NO** | **Name** | **Retention time（min）** | **Measured mass-charge ratio *m/z***） | **Molecular formula** | **Secondary mass spectrometry data** |
| --- | --- | --- | --- | --- | --- |
| 1 | nicotinamide | 1.21 | 123.0556 | C6H6N2O | 80.0495,53.0390 |
| 2 | hesperidin | 60.47 | 303.0869 | C16H14O6 | 267.0642,239.0700,213.0545,203.0698,185.0593,165.0694,129.0690,73.0281 |
| 3 | sappanone B | 60.47 | 303.0868 | C16H14O6 | 267.0640,239.0695,221.0589,213.0543,185.0590,175.0744,157.0642,129.0692,73.0280,55.0177 |
| 4 | protosappanin C | 60.47 | 303.087 | C16H14O6 | 267.0640,239.0693,221.0590,213.0545,203.0699,185.0590,175.0750,157.0643,129.0692,115.0535,73.0281,55.0177 |
| 5 | 2,2',5'-trihydroxy-4-methoxy chalcone | 17.98 | 287.092 | C16H14O5 | 229.0488,213.0543,185.0589,168.0693,157.0644,131.0490,91.0541,68.9972,55.0179 |
| 6 | senkyunolide | 66.69 | 209.1176 | C12H16O3 | 153.0542,105.0693,91.0540,79.0544,77.0385,55.0540 |
| 7 | senkyunolide G | 66.69 | 209.1175 | C12H16O3 | 129.0689,115.0525,107.0485,97.0642,91.0540,79.0542,77.0384,55.0540 |
| 8 | 4-hydroxy-3-butylphthalide | 58.81 | 207.1018 | C12H14O3 | 133.0642,117.0694,105.0695,91.0541,77.0385,65.0385,55.0177 |
| 9 | 3-(4-ethylbenzoyl) propionic acid | 58.81 | 207.1018 | C12H14O3 | 133.0641,117.0691,105.0694,91.0540,77.0385,67.0537,55.0541 |
| 10 | brazilein | 60.96 | 285.0758 | C16H12O5 | 239.3694,221.0591,197.0589,183.1794,147.0435,123.0438,102.0460,68.9972,55.0177 |
| 11 | albiflorin | 45.2 | 481.1721 | C23H28O11 | 151.0753,133.0649,121.0643,105.0337,97.0280,85.0285,77.0386 |
| 12 | sappanone B | 46.55 | 303.088 | C16H16O6 | 231.0669,230.0583,229.0511,216.0431,215.0354,213.0559,211.0407,159.0457,109.0298,73.0296 |
